# The impact of *Hnrnpl* deficiency on transcriptional patterns of developing muscle cells

**DOI:** 10.1002/2211-5463.70117

**Published:** 2025-09-13

**Authors:** Hannah R. Littel, Mekala Gunasekaran, Audrey L. Daugherty, Natalya M. Wells, Johnnie Turner, Christine C. Bruels, Christina A. Pacak, Isabelle Draper, Peter B. Kang

**Affiliations:** ^1^ Greg Marzolf Jr. Muscular Dystrophy Center and Department of Neurology University of Minnesota Medical School Minneapolis MN USA; ^2^ Molecular Cardiology Research Institute, Tufts Medical Center Boston MA USA; ^3^ Institute for Translational Neuroscience, University of Minnesota Minneapolis MN USA

**Keywords:** HnRNP L, nanopore transcriptomics, RNA sequencing, skeletal muscle, splicing regulation

## Abstract

Heterogeneous nuclear ribonucleoproteins (hnRNPs) bind to RNA, regulating gene expression and splicing. HnRNP L contributes to muscle development and the pathogenesis of myotonic dystrophy. We hypothesized that hnRNP L regulates muscle expression and splicing patterns. Using nanopore long‐read transcriptome sequencing and qPCR analyses, we investigated the impact of *Hnrnpl* knockdown on myoblasts and knockdown of the orthologous gene *smooth* in *Drosophila*. Notch signaling genes and muscle‐related genes were dysregulated in both models. Several genes had altered splicing patterns, including *Lamp2*, *Fhl1*, and *Dtna*. The α‐DB1 isoform of Dtna was downregulated, whereas the α‐DB3 isoform was upregulated. Our findings indicate that hnRNP L regulates both the transcription levels and splicing patterns of genes relevant to skeletal muscle development. We demonstrate the capabilities of long‐read transcriptome sequencing to study muscle development. Comparisons between nanopore long‐read transcriptome sequencing and data from PCR and qPCR analyses suggest that a minimum read depth of 10 is needed on nanopore sequencing to detect splicing differences greater than 10% to 20%. Future studies could determine whether the minimum read depth that we identified in our model is valid across a broader range of genes, cell types, and conditions. There are also intriguing hints of therapeutic implications of hnRNP L regulation for muscle diseases that merit further investigation.

## Graphical abstract

We performed nanopore whole‐transcriptome sequencing comparing RNA from *Hnrnpl*‐knockdown versus control C2C12 myoblasts to investigate the contributions of *Hnrnpl* to muscle development. Our results indicate that *Hnrnpl* regulates the expression of genes involved with Notch signaling and skeletal muscle, particularly splicing patterns of specific muscle genes. *Hnrnpl* also regulates apoptosis, reactive oxygen species (ROS) accumulation, and mitochondrial function.

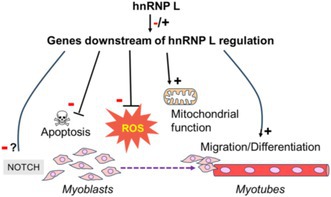

**Abbreviations**
CMA: chaperone‐mediated autophagy
DM1: myotonic dystrophy type 1
hnRNP L: heterogeneous nuclear ribonucleoprotein L
hnRNP LL: hnRNP L‐like
hnRNPs: heterogeneous nuclear ribonucleoproteins
IGV: integrative genome viewer
LOF: loss of function
LRT: likelihood‐ratio‐test
RBPs: RNA binding proteins
Sm: smooth

Heterogeneous nuclear ribonucleoproteins (hnRNPs) are a superfamily of RNA binding proteins (RBPs) with diverse functions including alternative splicing, mRNA stabilization, and regulation of transcription and translation [1]. The hnRNPs have been linked to numerous diseases such as cancer [2, 3, 4, 5, 6, 7], amyotrophic lateral sclerosis (ALS) with or without frontotemporal dementia (FTD) [8, 9], Alzheimer's disease [10], spinal muscular atrophy (SMA) [11, 12, 13], and rare neurodevelopmental disorders [14]. Regulation of mRNA stability and splicing by hnRNPs is important during the stem cell differentiation process, and many hnRNPs have important roles in maintaining the self‐renewal capacity of stem cells [15].

Several hnRNPs have been linked to myogenesis and muscle development [16, 17]. For example, hnRNP A1 modulates the splicing of various muscle development‐related genes, such as myocyte enhancer factor 2C (*MEF2C*) [18]. HnRNP D, also known as AUF1, promotes the translation of MEF2C [19] and plays a role in regulating myogenesis in muscle stem cells by targeting certain checkpoint mRNAs for decay [20]. HnRNP A2/B1 controls muscle differentiation in regenerating myogenic cells through regulation of alternative splicing of other RNA binding proteins, including MBNL1, MBNL2, and RBFox2 [21]. Inhibition of hnRNP E1, also known as PCBP1, leads to defects in proliferation and differentiation in mouse skeletal muscle satellite cells. Variants in hnRNP E1 in mice have significant impacts on muscle growth [22]. Furthermore, mutations in either *HNRNPA1* or *HNRNPDL* can lead to muscular dystrophy in humans [23, 24, 25].

Similar to other hnRNPs, hnRNP L plays a role in muscle development. HnRNP L binds to the muscle‐specific long noncoding RNA lncFAM to form a complex that binds to the *MYBPC2* promoter to increase its transcription, thus promoting myogenesis [26]. The transcription factor MyoD activates the production of enhancer RNAs, which then bind to hnRNP L to regulate the expression of downstream genes such as myoglobin during skeletal myoblast differentiation [27]. HnRNP L binding sites have been identified on *JAG2* mRNA, which encodes a canonical Notch ligand, as well as on other transcripts of key partners involved in Notch signaling (e.g., *NOTCH2*, *NOTCH3*, *POGLUT1*, and *NUMB*) [2]. A role for hnRNP L in the regulation of Notch‐mediated muscle function was previously reported [28, 29]. Mutations in the canonical Notch ligand *JAG2*, as well as in two other Notch pathway genes (*POGLUT1* and *MEGF10*), cause muscle disease phenotypes [29]. HnRNP L acts antagonistically with hnRNP L‐like (hnRNP LL) in the splicing suppression of *CHRNA1*, which is associated with congenital myasthenic syndrome [30]. HnRNP L is also a binding partner of MBNL1, and together they play a role in the pathophysiology of myotonic dystrophy [31].

The link between muscle development and hnRNP L is conserved across species. Knockdown of the hnRNP L orthologs in flies and zebrafish leads to a muscle phenotype. Mutations in *smooth*, the *Drosophila* ortholog of hnRNP L, are associated with muscle degeneration and thoracic muscle defects in flies [32]. Knockdown of *Hnrnpl2*, the hnRNP L ortholog in zebrafish, leads to severe muscle abnormalities [31]. The complete knockout of *Hnrnpl* in mice results in embryonic lethality [33]. In humans, multiple pathogenic variants in the *HNRNPL* gene have been reported in GeneMatcher (e.g., LOF variants); however, the genotype–phenotype correlation remains to be confirmed. Recently, it was shown that hnRNP L plays a role in heart function [34]. Knockdown of *HNRNPL* in human primary myoblasts results in impaired myotube fusion during differentiation [31]. However, the extent to which hnRNP L regulates the transcriptome of the developing muscle is still unknown.

HnRNP L binds to CA‐repeats and CA‐rich clusters on its RNA targets to regulate their expression and/or splicing [35, 36]. Binding of hnRNP L can either activate or repress splicing [37]. The protein preferentially binds to 3′ UTRs and introns [38]. Alternative exons with weak 5′ splice sites are correlated with hnRNP L‐dependent splicing regulation [39]. In addition, hnRNP L regulates itself in a negative feedback loop [40].

In this study, we extend the investigation of the impact of hnRNP L knockdown in muscle by examining the transcriptional profile of *Hnrnpl* deficient C2C12 myoblasts. RNA from *Hnrnpl* shRNA and control shRNA C2C12 myoblasts was used to carry out nanopore RNA sequencing. In parallel *in vivo* studies, we knocked down the orthologous fly gene *smooth* (*sm*) in *Drosophila* to identify the myogenic stages that are sensitive to decreased levels of this evolutionarily conserved RBP.

## Materials and methods

### Cell culture

C2C12 myoblast cultures were established and maintained in growth medium containing DMEM (Corning, Corning, NY, USA) + 10% fetal bovine serum (FBS) (GenClone, El Cajon, CA, USA) + 1% penicillin/streptomycin (Gibco). To induce differentiation, the cells were then cultured in low serum medium supplemented with DMEM + 2% horse serum (Gibco, Waltham, MA, USA). The differentiation medium was replenished every 48 h. The C2C12 cells (CRL‐1772) were originally obtained from the American Type Culture Collection (ATCC, Manassas, VA, USA). Mapping of the RNAseq data to the mouse genome confirmed the identity of the cell lines and the absence of contamination within the RNA sample. Cell cultures were checked for standard signs of contamination, including unexpected color changes and odors, and none of these signs were observed in the cultures used for the experiments in this report.

### shRNA mediated gene knockdown

C2C12 myoblasts were transfected with either a cocktail of three different shRNAs targeting mouse‐*Hnrnpl* or scrambled shRNA control (catalog number MSH092806‐CU6, GeneCopoeia, Rockville, MD, USA) using Lipofectamine 3000 (Invitrogen, Waltham, MA, USA). The three shRNAs target the following sequences on mouse *Hnrnpl*—NM_177301.5:

*Hnrnpl* (1)—ATTGACGGAGTAGTGGAAGCT

*Hnrnpl* (2)—CTACTCCATAACCACGGATGT

*Hnrnpl* (3)—ATGGTCTATGGCTTGGATCAA

The plasmids containing the shRNA sequences also expressed an eGFP reporter. Clones expressing the eGFP reporter were isolated, and stable cells were selected using puromycin 3 μg·mL^−1^ (P8833, Sigma, Burlington, MA, USA). eGFP expression was detected using a Leica DM IL LED microscope (Leica, Wetzlar, Germany). The eGFP‐positive clones were expanded, the knockdown efficiency was evaluated with qPCR, and the cells were used for further experimentation. Replicates for each experiment were obtained from different passages of the same selected stable cell line.

### Cell counts

To assess cell proliferation, 5000 C2C12 myoblasts were seeded in duplicate on Day 0. The cells were stained with trypan blue using a 1 : 1 dilution on Days 1, 2, and 3, then counted using a hemocytometer.

### Myotube fusion index

C2C12 myoblasts were grown in normal growth medium of DMEM containing 10% FBS and 1% penicillin–streptomycin on coverslips to 80% confluence. Then, the cells were switched to differentiation medium with 2% horse serum. On Day 7, the cells were fixed in 4% paraformaldehyde (PFA) for 15 min and blocked in serum medium for 1 h. The cells were stained with Rb‐Desmin (ab8592, Abcam, Waltham, MA, USA) primary antibody for 1 h. Cells were then stained with anti‐rabbit Alexa Fluor‐568 secondary (Invitrogen, A‐11036) antibody for 1 h. Coverslips were mounted using ProLong Gold Antifade Mountant with DAPI (Invitrogen). The slides were imaged using a DM6000B or DM5500B epifluorescent microscope (Leica). The myotube fusion index was determined by counting the number of nuclei within Desmin‐positive myotubes divided by the total number of nuclei in the field of view using imagej (National Institutes of Health, Bethesda, Maryland).

### Taqman notch plate array

The differential expression of Notch pathway genes was analyzed using a Taqman Notch plate array as previously described [41]. The Notch plate array included *Rn18s*, *Gapdh*, and *Actb* as endogenous controls. Ultimately, *Gapdh* was chosen for the ΔΔ*C*_t_ analyses because it was the most consistent across the plates.

### Nanopore long read transcriptome sequence (RNAseq) generation

RNA was extracted from two biological replicates each of scrambled and *Hnrnpl* shRNA‐treated C2C12 myoblasts using the PureLink RNA Mini Kit (Invitrogen). RNA concentration and purity were verified using a NanodropOne Spectrophotometer (Thermo Fisher Scientific, Waltham, MA, USA), and RNA integrity was determined using a 2100 Bioanalyzer (Agilent, Santa Clara, CA, USA). 1 μg of total RNA was used for nanopore direct RNA sequencing (SQK‐RNA002) on R9.4.1 flow cells using a GridION sequencer (Oxford Nanopore Technologies, Oxford, UK). Of note, the SQK‐RNA002 kit and the R9.4.1 flow cells are no longer available from Oxford Nanopore Technologies.

### RNAseq data analysis

RNAseq data were basecalled using guppy v.6.0.1, and bases called were counted with nanoplot v1.32.1. Basecalled data were mapped to the mouse genome (GRCm39) using splice‐aware minimap2 v.2.17, and the number of bases and reads mapped were counted with samtools stats v.1.9. Gene transcript numbers were counted using htseq v.0.9.1. Specific transcript variants were estimated using nanocount v1.1.0. Transcripts were visualized and manually counted using Integrative Genome Viewer (igv) v.2.16.1. Using rstudio Server (4.4.0‐openblas), the package deseq2 v.1.42.1 was used for differential gene expression analysis on the transcript counts produced by htseq. In deseq2, the results of the Wald significance test (default) were compared to the results of the Likelihood‐Ratio Test (LRT). A parametric fit (default) versus a local fit were also compared for the fitting of dispersions. The default method was used for size factor estimation (sfType = ratio), and *P*‐adjustment method (BH). Genes with a baseMean < 10 or a |log2FC| < 0.5 were filtered out of the list of significant genes. We evaluated splicing changes using two complementary r packages, isoformswitchanalyzer and dexseq. isoformswitchanalyzer v.2.2.0 was used to calculate differential isoform usage on the transcript variant counts estimated by nanocount, as detailed in Gleeson *et al*. [42]. The gene expression cut off was set at 10 while the isoform expression cut off was set at 5. dexseq v1.48.0 was used to evaluate differential exon usage based on the mapped alignment files produced by minimap2. Exonic regions with an exonBaseMean < 10 or a |log2FC| < 0.5 were filtered out of the list of significant exons. An adjusted *P* value < 0.05 was determined to be significant for the computational analyses.

### PCR and qPCR

RNAseq findings were validated using PCR and/or qPCR. 500 ng of RNA was converted to cDNA using the High Capacity RNA‐to‐cDNA kit (Applied Biosystems, Waltham, MA, USA). The cDNA was diluted by a factor of 10 before being used for qPCR. All Taqman qPCR probes (Thermofisher) used are listed in Table S1. The reaction was run using Taqman Fast Advanced Master Mix (Thermofisher) in the quantstudio 3 (Applied Biosystems) thermal cycler. Fold changes were calculated using the ΔΔ*C*_t_ method, normalized to *Gapdh*. For qPCR validation, the average of three technical triplicates was calculated for five biological replicates. For PCR, the cDNA was diluted with nuclease‐free water by a factor of 5, 10, or 20. The same dilution was used when comparing different transcripts of the same gene. The three biological replicates were used for PCR confirmation, and the PCR was repeated three times. The housekeeping genes *Gapdh* and *Actb* were also amplified at every dilution as a control. Primers used for PCR are listed in Table S2. PCR products were equally loaded onto an agarose gel, and imagej was used to calculate the band intensities on the inverted image. All statistical analyses were carried out using graphpad prism 10.3.0.

### *Drosophila* stocks and culture

The Gal4 driver lines [43] were purchased from the Bloomington Drosophila Stock Center (Indiana University, Bloomington, IN, USA). The corresponding fly line genotypes are indicated in Fig. 7. All strains were raised at 25 °C in a 12‐h light/12‐h dark cycle on standard *Drosophila* media. To generate flies that downregulate *smooth*, transgenics carrying the *UAS‐sm* IR (Inverted Repeat) transgene were crossed with Gal4 driver flies at 29 °C. The number of adult experimental progeny (i.e., double transgenics *sm* RNAi flies) and control progeny (i.e., single transgenic siblings that emerge in the same vial as the *sm* RNAi flies) was recorded for each cross. Of note, the Gal4/UAS system is less efficient at a lower temperature (i.e., 23 °C), thus enabling the generation of an increased number of ‘escaper’ *sm* RNAi progeny for molecular/cellular analysis. The *Mef2Gal4* muscle‐specific driver [44] was used at 23 °C to generate the corresponding *smooth* knockdown and control male flies for further RNA analysis.

### RNA extraction and transcriptome analysis from *smooth* knockdown *Drosophila*

Total RNA was isolated from 20 to 40 flies or approximately 35 mg of thorax tissue [45] from *smooth* knockdown flies (Mef2Gal4 > *smooth* RNAi) and corresponding control flies (Mef4Gal4;cy control siblings). RNA concentration and purity were verified using a NanodropOne Spectrophotometer (Thermofisher Scientific). 500 ng of RNA was reverse transcribed into cDNA using the High Capacity RNA to cDNA Kit (Applied Biosystems). The cDNA product was used for qPCR with probes for *smooth*, as well as other genes of interest including *draper*, *Serrate*, *rumi*, *Notch*, *misfire*, and *Limpet* (Table S1). The reaction was run using Taqman Fast Advanced Master Mix (Thermofisher) in the quantstudio 3 (Applied Biosystems) thermal cycler. *Act5c* amplification on the same cDNA was used as a control to calculate fold change of transcripts using the ΔΔ*C*_t_ method.

## Results

### Knockdown of *Hnrnpl* via shRNA

**Fig. 1:**
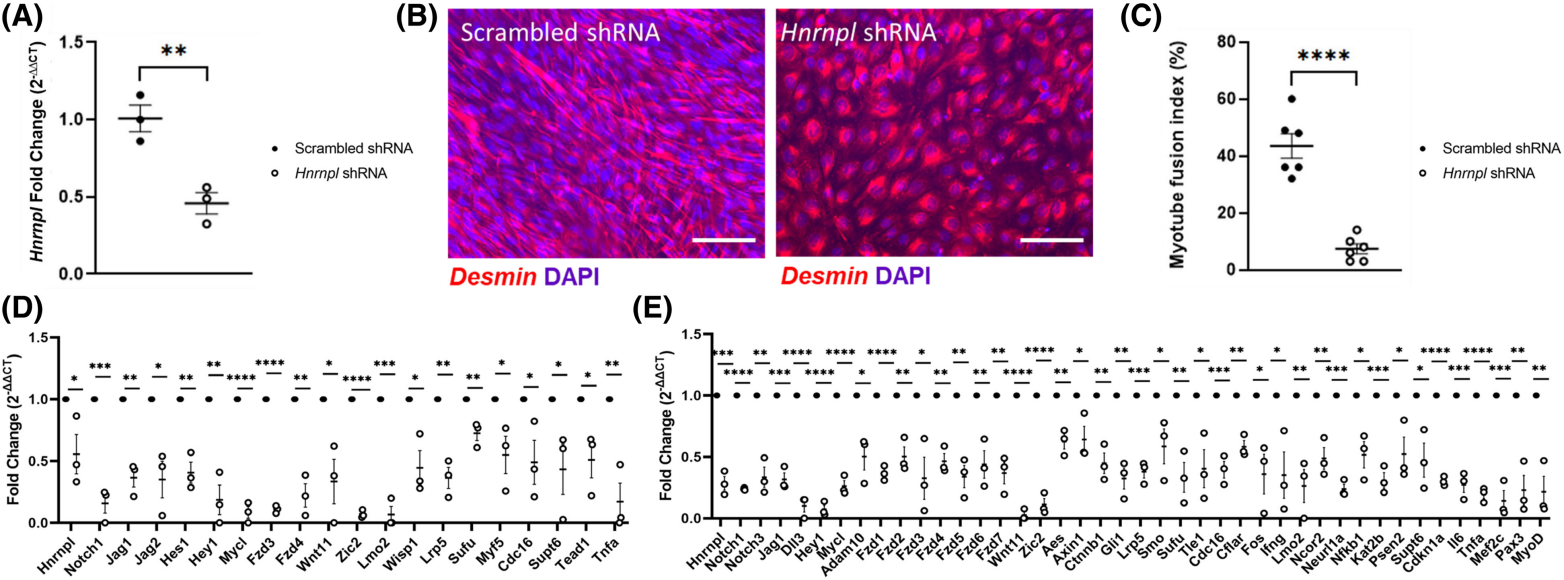
*Hnrnpl* knockdown in C2C12 cells. (A) Knockdown efficiency of *Hnrnpl* was assessed by qPCR. Results are from *N* = 3 replicates. (B) Scrambled and *Hnrnpl* shRNA‐treated cells were differentiated for 7 days in low serum medium. Myogenic potential was found to be downregulated in *Hnrnpl* knockdown condition. Representative immunofluorescence images of Desmin‐stained scrambled vs. *Hnrnpl* knockdown cells are shown. Two different fields of view were analyzed from three different replicates for 6 data points total. Scale bar, 100 μm. (C) Myotube fusion index assay on the scrambled and *Hnnrpl* knockdown cells. Taqman plate arrays of Notch genes run on myoblast (D) and myotube (E) stage cells showing several important Notch pathway genes being significantly downregulated in the *Hnrnpl* knockdown condition. Results are from *N* = 3 replicates. Transcript levels were normalized to *Gapdh* using ΔΔ*C*_t_ method. Unpaired *T*‐tests were used for significance: *, *P* < 0.05; **, *P* < 0.01; ***, *P* < 0.001; ****, *P* < 0.0001. Error bars show standard error of the mean (SEM).

We transfected C2C12 myoblasts with *Hnrnpl* shRNA and verified the knockdown efficiency to be approximately 50% in myoblasts (Fig. 1A). We found that *Hnrnpl* knockdown had no effect on proliferation (Fig. S1) but did impact differentiation. Specifically, the myotube fusion index was reduced in the cells treated with *Hnrnpl* shRNA (Fig. 1B,C). Our proliferation and differentiation results in C2C12 myoblasts mirror previous results from lentiviral *HNRNPL* knockdown in human primary myoblasts [31]. The knockdown efficiency of *Hnrnpl* in the differentiated myotubes increased to approximately 70% (Fig. 1E).

### Effect of *Hnrnpl* knockdown on notch pathway genes

Since *HNRNPL* has known binding sites on the Notch ligand *JAG2* mRNA as well as on other key Notch pathway gene transcripts [2], we investigated the effect of *Hnrnpl* knockdown on Notch signaling pathway gene expression, in both myoblasts (Fig. 1D) and myotubes (Fig. 1E). Nineteen Notch pathway genes were significantly downregulated at the myoblast stage, and 41 genes were significantly downregulated at the myotube stage, excluding *Hnrnpl*. No genes were significantly upregulated at either stage (Table S3).

We compared the deseq2 results from nanopore RNA sequencing to the Notch pathway qPCR results. In general, deseq2 predicted a similar fold change to that found on qPCR, with a Pearson's correlation coefficient of 0.5215 (Table S3, Fig. S2). Many of the Notch pathway expression changes that were significant on qPCR were not significant on deseq2, likely due to low coverage (low baseMean). Forty‐one out of 96 genes on the Notch plate array had baseMean values below the analysis cut off of 10 on deseq2 analysis (Table S3), indicating that those genes had low sequencing coverage and likely low myoblast expression. Of the 19 Notch plate array genes that were significantly downregulated on qPCR on RNA derived from myoblasts (Fig. 1D), nine had a baseMean less than 10 on deseq2 (*Notch1*, *Hey1*, *Mycl*, *Fzd3*, *Fzd4*, *Wnt11*, *Zic2*, *Lmo2*, and *Tnfa*), so adjusted *P*‐values were not calculated for those genes (Table S3). qPCR is likely to be more sensitive to genes with low expression than nanopore RNAseq.

Due to the low coverage of many Notch genes, splicing patterns of Notch genes in the *Hnrnpl* knockdown cells could not be analyzed via nanopore RNAseq. We performed PCR analyses to investigate the possibility of splicing changes in Notch genes, but the results were inconclusive due to variability (data not shown). The spectrum of putative hnRNP L‐induced splicing changes in Notch genes remains to be defined using PCR probes that cover alternatively spliced and cryptic exon candidates in the corresponding gene loci.

### Differential gene expression analysis

**Fig. 2:**
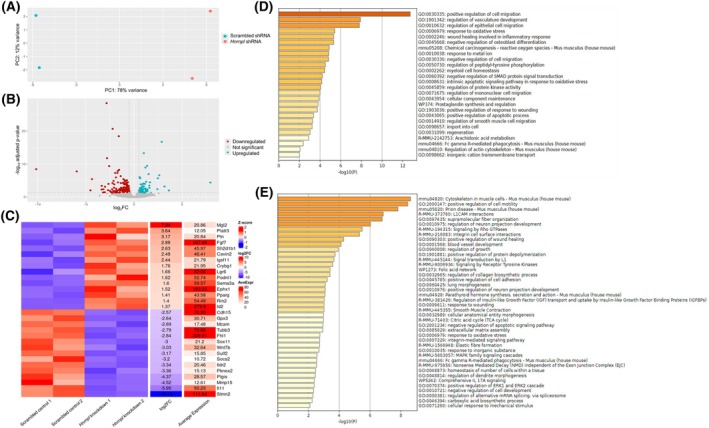
deseq2 analysis of nanopore RNA sequence data. (A) R‐log transformed PC plot of the gene counts, using a local dispersion fit. (B) Volcano plot of all 8507 genes with an adjusted *P*‐value from deseq2. Genes found to be significant (*P*adj < 0.05; |log2FC| > 0.5) using the Wald test and local fit are in color. 80 genes were upregulated (teal), while 148 genes were downregulated (red). (C) Heatmap of the top 15 most upregulated and downregulated genes found by deseq2, sorted by log_2_FC. (D) Top gene enrichment clusters of the significantly upregulated genes. (E) Top gene enrichment clusters of the significantly downregulated genes. Gene enrichment clusters were generated using metascape v3.5.20240219 [46].

After performing nanopore RNAseq, each sample had approximately 2 billion bases that mapped to the mouse genome (Table S4). The number of each gene transcript was counted and input into deseq2 for differential gene expression analysis. The scrambled and *Hnrnpl* knockdown groups clearly separated on a principal component analysis (PCA) plot (Fig. 2A). Two statistical tests were used: the Wald significance test and the Likelihood‐Ratio‐Test (LRT). Each test was run using a parametric fit (default) and a local fit. Thus, the analysis was performed using four different configurations. Using the Wald test and a local fit yielded the highest number of significant genes (Table S5). We performed qPCR on five splicing factors to validate the results of the four different configurations. The results of the Wald test with a local fit were most similar to the qPCR results (Table 1), so these parameters were used for downstream analysis. The statistically significant results of all four different analyses are shown in Tables S6–S10.

**Table 1:** qPCR analysis and comparison to deseq2. Results of deseq2 analyses for several different splicing factors calculated using 4 different methods: the Wald test + parametric fit (W para) (the default method), LRT + parametric fit (LRT para), Wald test + local fit (W local), and LRT + local fit (LRT local). The fold change (FC) between the local and parametric fit was the same when looking at the first three decimal places. The fold change and adjusted *P*‐values (*P*adj) from deseq2 were compared to the fold changes and *P*‐values (*P*val) from qPCR validation using *N* = 5 replicates. Statistical analysis of qPCR data was performed using an unpaired *T*‐test in graphpad prism.

Gene	*Hnrnpl*	*Hnrnpk*	*Hnrnpa2b1*	*Mbnl1*	*Mbnl2*
deseq2 FC	0.581	1.402	1.294	1.377	0.487
W para *P*adj	0.0567	0.2444	0.3213	0.2293	0.0022
LRT para *P*adj	0.0670	0.2777	0.3626	0.2586	0.0027
W local *P*adj	0.0088	0.0527	0.0784	0.0486	6.08E‐05
LRT local *P*adj	0.0095	0.0566	0.0842	0.0511	6.51E‐05
qPCR FC	0.537	0.869	0.985	1.298	0.432
qPCR *P*val	< 0.0001	0.0145	0.8626	< 0.0001	< 0.0001

With the Wald test and local fit, there were a total of 8507 detected genes with an adjusted *P*‐value from deseq2. Eighty genes were significantly upregulated, and 148 genes were significantly downregulated (Fig. 2B). Among these, *Mgl2* was the most upregulated gene (log_2_fold change of 7.9), and *Stmn2* was the most downregulated gene (log_2_fold change of −10) (Fig. 2C). Two enriched upregulated pathways were regulation of apoptosis and regulation of cell migration (Fig. 2D). Several downregulated pathways were notable, including cytoskeleton in muscle cells, muscle contraction, regulation of neuron projection development, regulation of collagen biosynthesis, negative regulation of apoptotic pathways, and regulation of alternative splicing via the spliceosome (Fig. 2E). In addition, response to oxidative stress and wound healing appeared in the enriched pathways for both the up and downregulated genes.

**Fig. 3:**
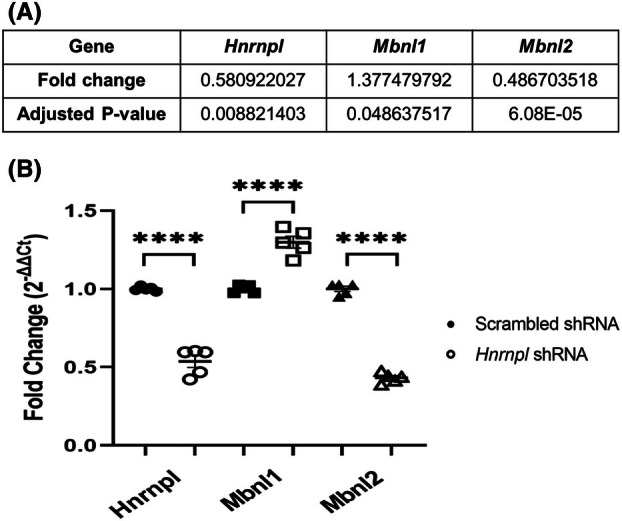
Confirmation of deseq2 results. (A) DESeq2 results of three different splicing factors calculated using the Wald test with a local fit. (B) qPCR verification of three of the splicing factors that corresponded with the deseq2 predictions. Transcript levels were normalized to Gapdh using ΔΔ*C*_t_ method. *Hnrnpl*, *Mbnl1*, and *Mbnl2* were all significant using an unpaired *T*‐test (*P* < 0.0001 for all). Mean fold changes are 0.5368, 1.298, and 0.4318 respectively. *N* = 5 replicates. Error bars show standard error of the mean (SEM).

On deseq2 analysis, the splicing factor *Mbnl2* was significantly downregulated. For *Mbnl1*, the log_2_FC was less than the cutoff of 0.5, but it was significantly upregulated after *P*‐adjustment (Fig. 3A). We decided to confirm the expression levels of these splicing factors due to their links to myotonic dystrophy [47, 48, 49]. Using qPCR, we verified the deseq2 results for *Hnrnpl*, *Mbnl1*, and *Mbnl2* (Fig. 3B). *Mbnl1* had an average fold change of 1.3, while *Mbnl2* had an average fold change of 0.4. Both changes were statistically significant. We examined whether the expression of other hnRNP genes was altered by *Hnrnpl* knockdown. deseq2 found no significant changes in expression of any hnRNP gene assessed, including *Hnrnpll* (hnRNP L‐like) (Table S11). *Hnrnpk* and *Hnrnpa2b1* were close to being significant; however, the qPCR results for these genes did not confirm the deseq2 fold change (Table 1).

### Differential splicing analysis

We used two complementary approaches to splicing analysis. First, we quantified specific transcript variants using nanocount [42], and then analyzed those counts in isoformswitchanalyzer, which yielded 23 significantly differentially expressed transcripts representing 14 different genes (Table S12). Next, we quantified differential exon usage with dexseq and found 132 significant exonic regions in 97 distinct genes (Table S13). Five alternatively spliced genes appeared in both the isoformswitchanalyzer and dexseq analyses: *Aqp5*, *Cryab*, *Lamp2*, *Pdlim7*, and *Tpm1* (Table S14). *Aqp5* and *Pdlim7* were not analyzed further because they have not been previously reported to cause a muscle disease phenotype. *CRYAB* has been linked to muscle disease [50, 51]; however, splicing differences in *Cryab* would not result in any change on the protein level, so the functional effect of alternative splicing would be more difficult to elucidate.

**Fig. 4:**
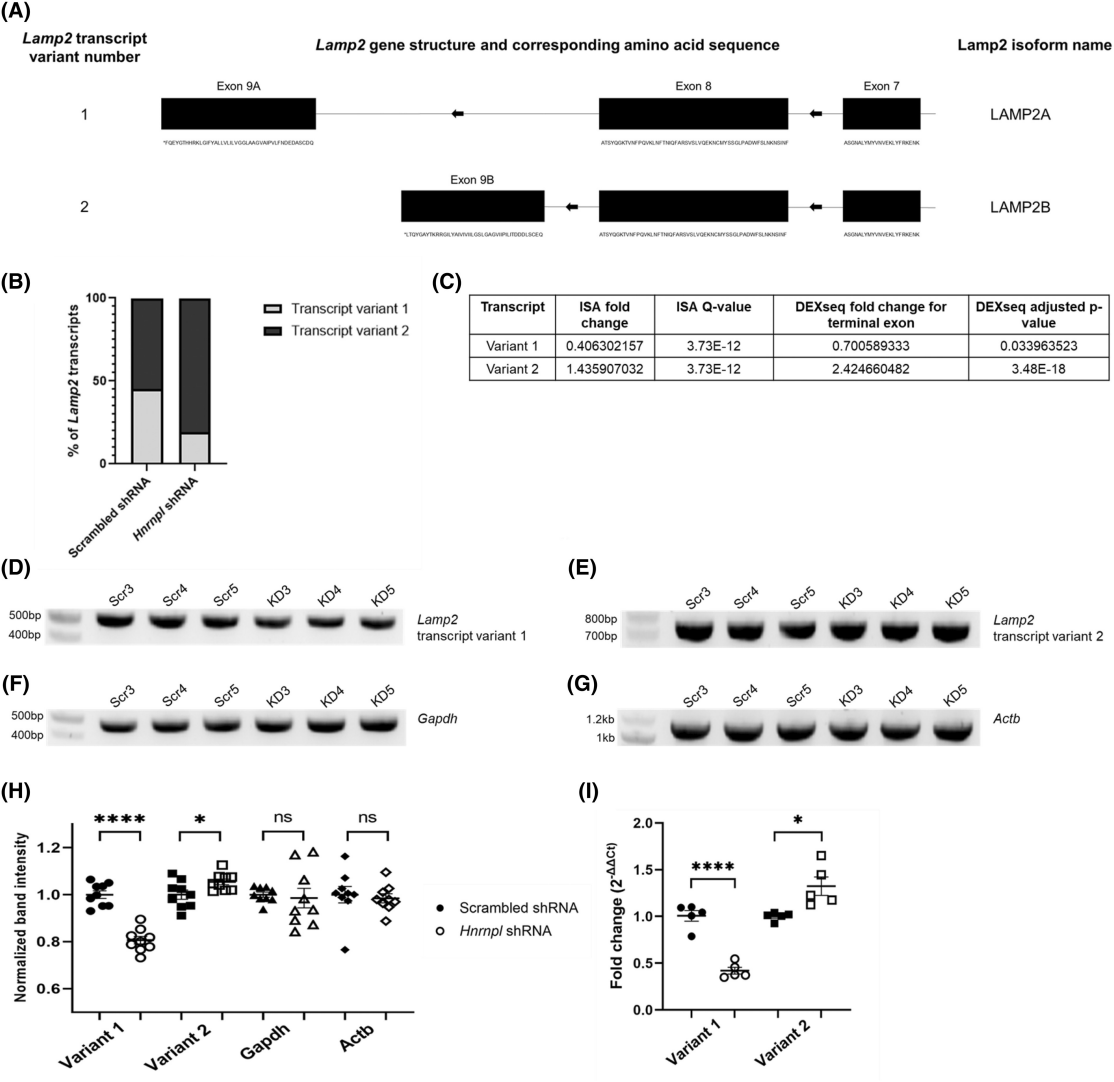
Splicing change in *Lamp2* in C2C12 myoblasts transfected with scrambled shRNA (Scr) or *Hnrnpl* shRNA (KD). (A) Schematic of the *Lamp2* transcripts of interest. The *Lamp2* coding sequence is on the reverse strand. *Lamp2* transcript variant 3 is not shown, as it had no expression seen in the nanopore data. (B) Comparing the percentage of nanopore reads mapping to *Lamp2* transcript variant 1 and *Lamp2* transcript variant 2 between the scrambled and *Hnrnpl* shRNA groups. Total number of reads mapped to *Lamp2* was calculated with HTSeq. Reads mapped to transcript‐specific terminal exons were counted by dexseq. (C) Results from isoformswitchanalyzer (ISA) and dexseq for *Lamp2*. The *Q*‐value is the FDR corrected *P*‐value. (D) PCR confirmation of *Lamp2* transcript variant 1 (512 bp) and (E) *Lamp2* transcript variant 2 (742 bp). Housekeeping genes (F) *Gapdh* (467 bp) and (G) *Actb* (1115 bp) are shown. 500 ng of RNA from 3 replicates of both scrambled (Scr 3–5) and *Hnrnpl* knockdown (KD 3–5) cells, independent from the replicates that were used for nanopore sequencing, were converted to cDNA, and the resulting cDNA was diluted by a factor of 20 before being used as a template for PCR. The PCRs were repeated in three triplicates and were run on separate gels. (H) Normalized imagej band intensity calculations of PCR products shown in (D–G). The 9 data points represent three technical triplicates from *n* = 3 independent replicates. The band intensities were normalized to the average scrambled band intensity. Results of an unpaired *T*‐test show that band intensities between the scrambled and *Hnrnpl* knockdown cells are significantly different for *Lamp2* transcript variant 1 (*P* < 0.0001) and *Lamp2* transcript variant 2 (*P* = 0.0302). *Gapdh* and *Actb* controls were amplified using the same cDNA dilution as a control (*P* = 0.7399 and *P* = 0.6998, respectively). (I) qPCR confirmation of *Lamp2* transcript variant 1 downregulation (*P* < 0.0001) and transcript variant 2 upregulation (*P* = 0.0120). Transcript levels were normalized to *Gapdh* using ΔΔ*C*_t_ method. *N* = 5 replicates. Unpaired *T*‐tests were used for significance. Error bars show standard error of the mean (SEM).

*Lamp2* displayed differential splicing of transcript variants 1 and 2 by both isoformswitchanalyzer and dexseq. *LAMP2* is associated with Danon disease in humans, which is a dominant X‐linked disorder clinically characterized by hypertrophic cardiomyopathy, skeletal myopathy, and intellectual disability [52]. *Lamp2* transcript variants 1 and 2 vary in their terminal exons and have different expression patterns (Fig. 4A). Transcript variant 1 (NM_001017959.2) was predicted to be downregulated, while transcript variant 2 (NM_010685.4) was predicted to be upregulated (Fig. 4B,C). In the nanopore data, 200–400 reads mapped to *Lamp2* in each sample. The *Lamp2* transcript composition of the scrambled samples was ~ 45% reads mapping to transcript variant 1 and ~ 55% to transcript variant 2; for the *Hnrnpl*‐shRNA treated samples, the composition was ~ 20% transcript variant 1 and ~ 80% transcript variant 2 (Table S15). PCR confirmed significant *Lamp2* transcript variant 1 downregulation and significant *Lamp2* transcript variant 2 upregulation (Fig. 4D–H). The findings were also validated on qPCR (Fig. 4I).

*Tpm1* underwent significant differential splicing in the setting of *Hnrnpl* knockdown that was detected by both isoformswitchanalyzer and dexseq (Table S14), and the overall expression level was significantly downregulated in all four deseq2 analyses (Table S10). *TPM1* is linked to several cardiac muscle phenotypes such as dilated cardiomyopathy [53], hypertrophic cardiomyopathy [54], and left ventricular noncompaction [55] in humans. A targeted analysis of the nanopore sequence data that was restricted to *Tpm1* was confirmed via qPCR and published as part of a previous study [34].

**Fig. 5:**
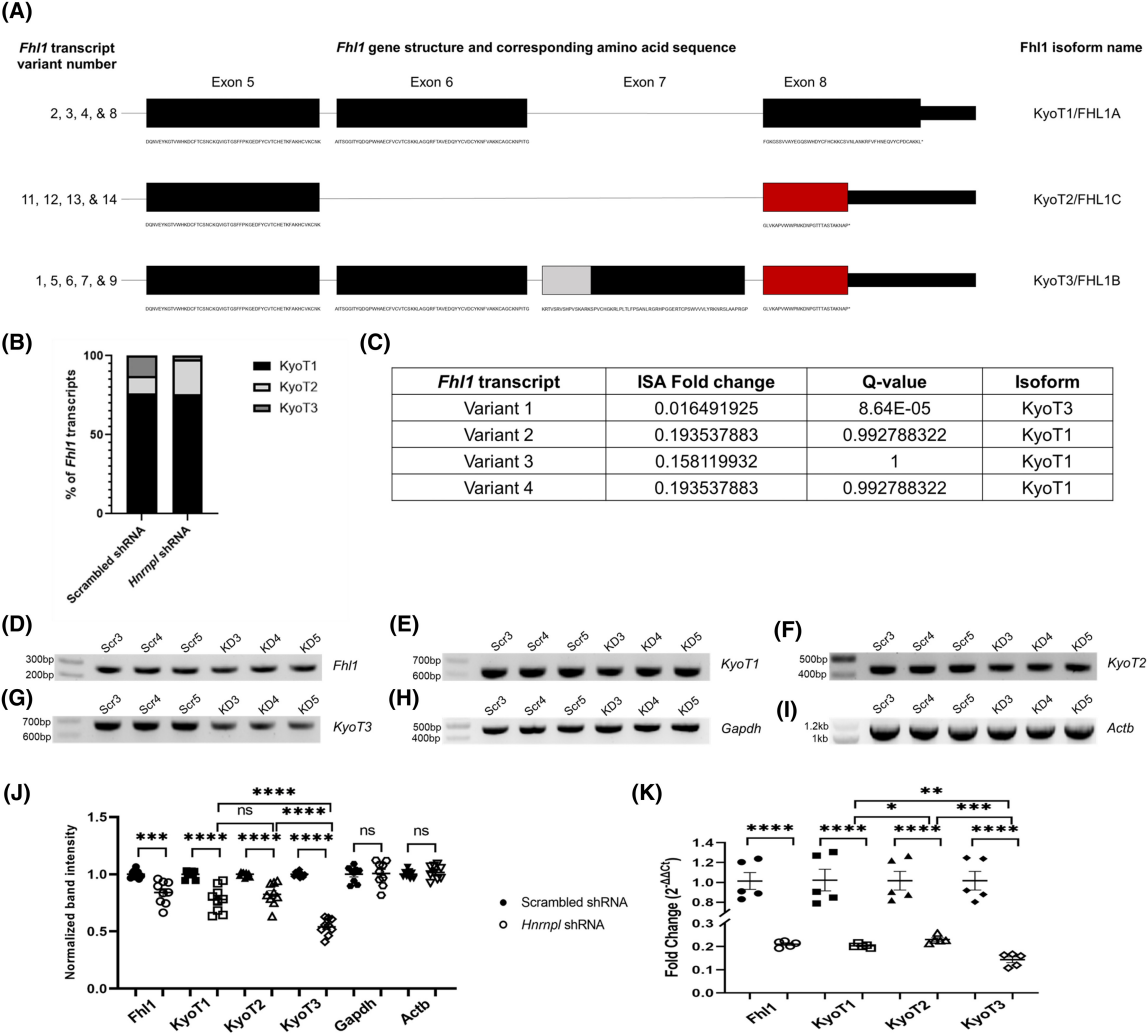
Differential downregulation of *Fhl1* transcripts in C2C12 myoblasts transfected with scrambled shRNA (Scr) or *Hnrnpl* shRNA (KD). (A) Schematic of the different *Fhl1* transcripts and which isoforms they correspond to. The gray box represents a nuclear localization signal (NLS), and the red box represents an RBP‐J‐binding motif. (B) Comparing the percentage of nanopore reads mapping to the different *Fhl1* isoforms between the scrambled and *Hnrnpl* shRNA groups. Total number of reads mapped to *Fhl1* was calculated with HTSeq. Reads mapped to specific isoforms were counted manually in igv. (C) isoformswitchanalyzer (ISA) results for *Fhl1*. The *Q*‐value is the FDR corrected *P*‐value. (D) PCR confirmation of *Fhl1* present in every transcript (267 bp), (E) transcripts encoding the KyoT1 isoform (628 bp), (F) transcripts encoding the KyoT2 isoform (439 bp), and (G) transcripts encoding the KyoT3 isoform (669 bp). Housekeeping genes (H) *Gapdh* (467 bp) and (I) *Actb* (1115 bp) are shown. 500 ng of RNA from 3 replicates of both scrambled (Scr 3–5) and *Hnrnpl* knockdown (KD 3–5) cells, independent from the replicates that were used for nanopore sequencing, were converted to cDNA, and the resulting cDNA was diluted by a factor of 10 before being used as a template for PCR. The PCRs were repeated in three triplicates and ran on separate gels. (J) Normalized imagej band intensity calculations of PCR products shown in (D–I). The 9 data points represent three technical triplicates from *n* = 3 independent replicates. The band intensities were normalized to the average scrambled band intensity. Results of an unpaired *T*‐test show that band intensities between the scrambled and *Hnrnpl* knockdown cells are significantly different for transcripts encoding all Fhl1 isoforms. The normalized band intensities between *Hnrnpl* knockdown KyoT1 vs. KyoT3, as well as KyoT2 vs. KyoT3 are also significantly different, but not KyoT1 vs. KyoT2. *Gapdh* and *Actb* controls were amplified using the same cDNA dilution as a control. (K) qPCR confirmation of the downregulation. Transcript levels were normalized to *Gapdh* using ΔΔ*C*_t_ method. *N* = 5 replicates used. Downregulation of *Fhl1* overall, KyoT1, KyoT2, and KyoT3 were all significant. The fold changes for the *Hnrnpl* knockdown KyoT1 vs. KyoT2, KyoT1 vs. KyoT3, and KyoT2 vs. KyoT3 were significant as well. Unpaired *T*‐tests were used for significance: *, *P* < 0.05; **, *P* < 0.01; ***, *P* < 0.001; ****, *P* < 0.0001. Error bars show standard error of the mean (SEM).

*Fhl1* was significantly downregulated on all four DESeq2 comparisons (Table S10). *Fhl1* variant expression levels were verified because it is linked to several inherited muscle diseases in humans [56, 57, 58, 59, 60]. The Fhl1 protein has three different predominant isoforms, KyoT1, KyoT2, and KyoT3. KyoT2 and KyoT3 both have an RBP‐J binding motif, and are corepressors for the Notch signaling pathway (Fig. 5A) [61, 62]. Transcripts encoding all three isoforms could be seen in the nanopore data, but transcripts encoding the KyoT3 isoform appeared in a lower proportion in the *Hnrnpl* knockdown than in the scrambled (Fig. 5B). Approximately 400 reads mapped in *Fhl1* in each scrambled sample, while about 50 reads mapped to *Fhl1* in each *Hnrnpl* knockdown sample. The composition of KyoT3 encoding transcripts switched from ~ 12–13% of reads in the scrambled samples to 0–4% of reads in the *Hnrnpl*‐shRNA‐treated samples (Table S14). isoformswitchanalyzer, but not dexseq, predicted differential regulation of *Fhl1* transcripts in the setting of *Hnrnpl* knockdown (Fig. 5C; Table S16). isoformswitchanalyzer only examined transcripts encoding KyoT1 and KyoT3, finding that while both were downregulated, transcripts encoding KyoT3 were significantly more downregulated than transcripts encoding KyoT1. On PCR (Fig. 5D–J), we found that KyoT3 was significantly more downregulated than KyoT1 or KyoT2. The PCR results were confirmed on qPCR, with more specificity (Fig. 5K). KyoT2 was the least downregulated with a fold change of 0.22, followed closely by KyoT1 with a fold change of 0.20. While all *Fhl1* transcript variants were downregulated, transcripts encoding the KyoT3 isoform were significantly more downregulated than transcripts encoding the other two isoforms, with a fold change of 0.15.

**Fig. 6:**
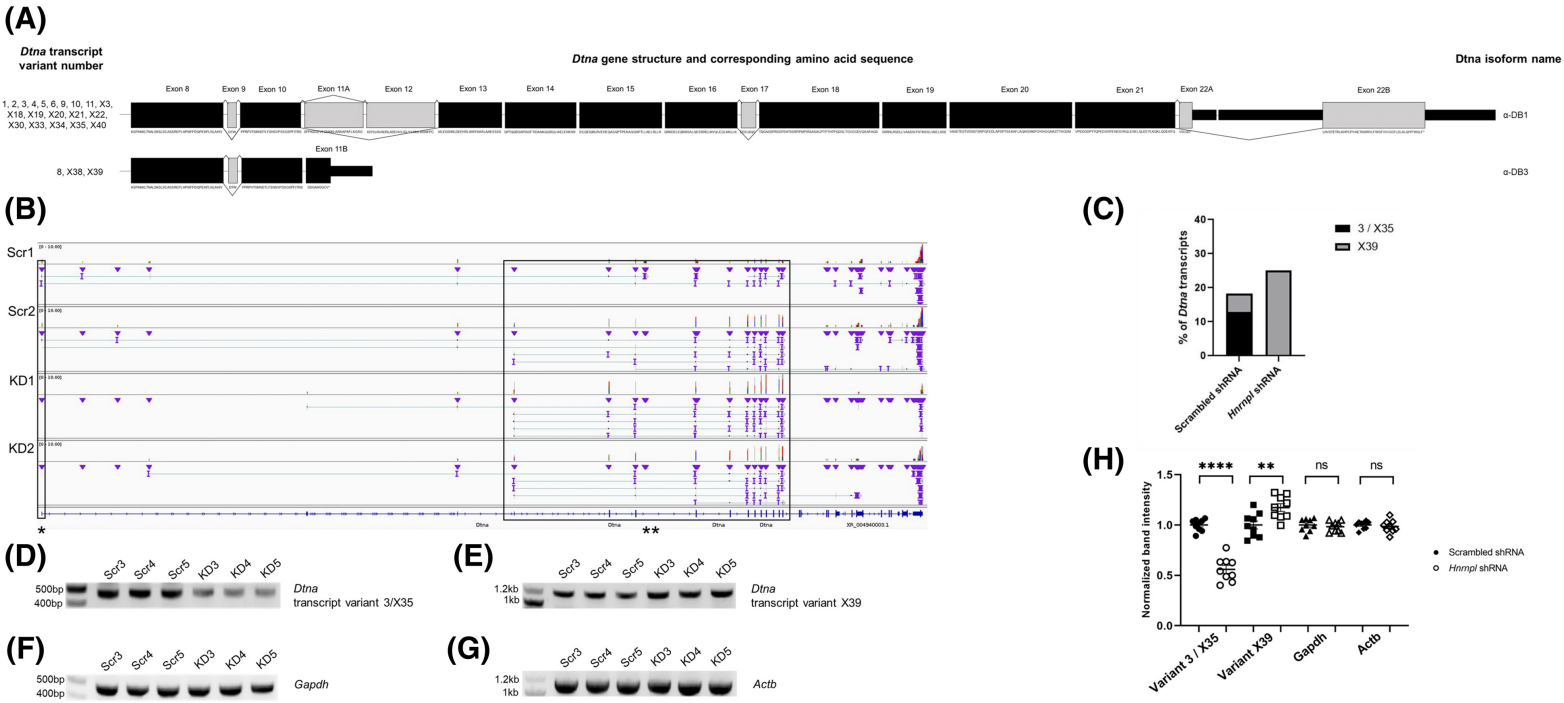
Splicing change in *Dtna* in C2C12 myoblasts transfected with scrambled shRNA (Scr) or *Hnrnpl* shRNA (KD). (A) Schematic of the *Dtna* transcripts and corresponding isoforms of interest. Isoform α‐DB2 was not a major product in the nanopore data, so it is not shown. Coding exons 1–8 are shared between the different isoforms, but there are several distinct noncoding exons which make the 5′ UTRs of the different transcripts unique (not shown). (B) Nanopore RNA sequencing coverage of *Dtna*, viewed in Integrated Genome Viewer (igv). The boxed exon marked with * is a noncoding start exon which is exclusive for *Dtna* transcript variant 3 (NM_001285813.1) and predicted transcript variant X35 (XM_030250320.2) at chr18:23443077‐23443328 mm39. The boxed transcript marked with ** is predicted *Dtna* transcript X39 (XM_036160981.1). (C) Comparing the percentage of nanopore reads mapping to the different *Dtna* transcripts between the scrambled and *Hnrnpl* shRNA groups. Total number of reads mapped to *Dtna* was calculated with HTSeq. Reads mapped to specific exons were counted manually in igv. Representative images of the PCR confirmation of (D) *Dtna* transcript variant 3/X35 (478 bp) and (E) predicted *Dtna* transcript variant X39 (1154 bp). Predicted transcript X39 was confirmed by Sanger sequencing (not shown). Housekeeping genes (F) *Gapdh* (467 bp) and (G) *Actb* (1115 bp) are shown. 500 ng of RNA from 3 replicates of both scrambled (Scr 3–5) and *Hnrnpl* knockdown (KD 3–5), independent from the replicates that were used for nanopore sequencing, were converted to cDNA, and the resulting cDNA was diluted by a factor of 5 before being used as a template for PCR. The PCRs were repeated in three triplicates and ran on separate gels. (H) Normalized imagej band intensity calculations of PCR products shown in (D–G). The 9 data points represent three technical triplicates from *n* = 3 independent replicates. The band intensities were normalized to the average scrambled band intensity. Results of an unpaired *T*‐test show that band intensities between the scrambled and *Hnrnpl* knockdown are significantly different for both *Dtna* transcript variant 3 (*P* < 0.0001) and *Dtna* transcript variant X39 (*P* = 0.0061). *Gapdh* and *Actb* controls were amplified using the same cDNA dilution as a control (*P* = 0.5377 and *P* = 0.5757, respectively). Error bars show standard error of the mean (SEM).

Previous bioinformatic analysis detected hnRNP L binding sites in *DTNA* [31]. Although splicing differences were not detected computationally in *Dtna*, we manually inspected the nanopore coverage of the gene and detected splice differences, which were then confirmed by PCR. *DTNA* has been linked to left ventricular noncompaction [63, 64], as well as a mild dominant form of muscular dystrophy [65]. An average of 16 reads mapped to *Dtna* in each sample in the nanopore sequencing data. In the scrambled samples, ~ 13% of reads mapped to chr18:23443077‐23443328 mm39, which is a noncoding start exon exclusive for transcript variant 3 (NM_001285813.1) and transcript variant X35 (XM_030250320.2) (referred to as transcript variant 3/X35 throughout the rest of the current report), while ~ 5% of reads mapped to transcript variant X39 (XM_036160981.1). In the *Hnrnpl*‐shRNA‐treated samples, none of the reads mapped to the start exon for transcript variant 3/X35, while 25% of the reads mapped to transcript variant X39 (Table S15). Neither isoformswitchanalyzer nor dexseq detected the findings, though the coverage in that region was suboptimal (Table S17). *Dtna* has many different transcript variants, which encode three predominant isoforms of different lengths (Fig. 6A). *Hnrnpl* knockdown led to diminished expression of transcript variant 3/X35, while reads mapping to predicted transcript X39 were increased (Fig. 6B,C). The downregulation of transcript 3/X35 and upregulation of transcript X39 was confirmed using PCR (Fig. 6D–H).

### Phenotype of *smooth* knockdown in *Drosophila*

**Fig. 7:**
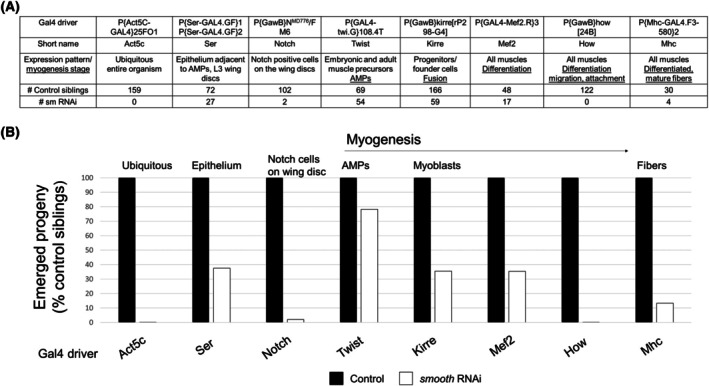
Myogenesis stage‐specific knockdown of *smooth* (*Hnrnpl*) in *Drosophila* indicates that *smooth* plays an important role at the stages of differentiation, pre‐fusing myoblasts and developing fibers, which coincide with decreasing Notch and twist activity. (A) Table showing different Gal4 drivers using for *smooth* knockdown, the expression pattern of the driver, and the number of control and *smooth* RNAi flies that emerged with each driver. (B) Bar graph representation of the percentage of *smooth* RNAi flies that emerged compared to control siblings with each driver.

Different Gal4 drivers were used to downregulate *smooth* either ubiquitously or at various stages of myogenesis, including in the Serrate‐positive cells that regulate muscle development [66]. Ubiquitous knockdown of *smooth* in *Drosophila* resulted in lethality, as was observed in *Hnrnpl* knockout mice [33]. The lethality phenotype was recapitulated when using the mesoderm‐specific *How‐Gal4* driver (AKA *24B‐Gal4*), indicating that *smooth*'s function is critical for muscle development. All drivers expressing the *smooth* RNAi transgene resulted in reduced adult eclosion (compared to the control siblings), although knockdown was more tolerated when targeting the adult muscle precursor (AMPs, using the *twist* driver) compared to later stages of myogenesis (Fig. 7). Altered migration of muscle cells (obtained with the *how* driver) could also contribute to the lethality of *smooth* RNAi flies (Fig. 7); notably, ‘migration’ was also highlighted in the pathway analysis of *Hnrnpl*‐deficient C2C12 cells (Fig. 2D). The *Mef2Gal4* driver promotes expression of the gene of interest in the embryonic mesoderm, which persists into adulthood [67, 68, 69], and it resulted in a workable population of knockdown progeny as compared to the *how* and *mhc* drivers (Fig. 7). The *kirre* and *twist* drivers produced more progeny than the *mef2* driver but only resulted in expression in founder cells (*kirre*) [70] or progenitor cells (*twist*) [71], so expression declines as myogenesis proceeds. Therefore, the *Mef2Gal4* driver was selected to generate the *Mef2Gal4 > smooth RNAi* male fly progeny used in the RNA analysis experiments. These flies displayed an age‐dependent ‘drooping’ wing phenotype, which was previously associated with muscle weakness in *Drosophila* [72].

### Differential gene expression in *Drosophila*

**Fig. 8:**
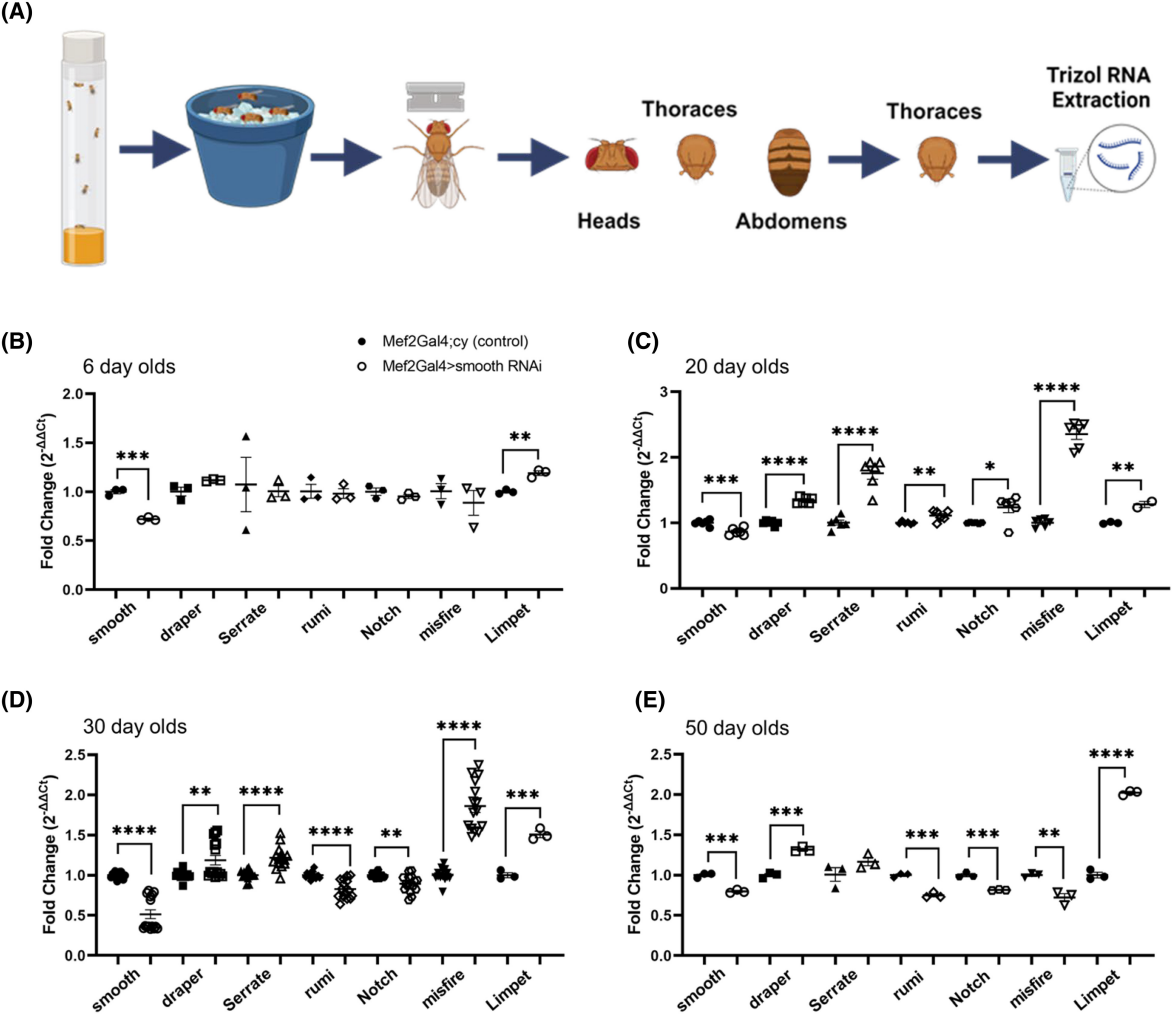
qPCR from *Drosophila* RNA. (A) Schematic showing RNA isolation from thoraces of Mef2Gal4 > *smooth* RNAi knockdown male *Drosophila* and corresponding Mef4Gal4;Cy driver control sibling *Drosophila*, and the qPCR results at ages (B) six, (C) 20, (D) 30, and (E) 50 days old. Transcript levels were normalized to *ACT5C* using the ΔΔ*C*_t_ method. Pooled fly samples are represented by technical replicates. Day 6 had three technical replicates per probe. Day 20 had six technical replicates per probe, except for *Limpet* which only had three technical replicates, and one *smooth* RNAi replicate was removed for being an outlier. Day 30 had 15 technical replicates per probe, across two different batches of flies. Day 50 had three technical replicates per probe. Unpaired *T*‐tests were used for significance between control and *smooth* RNAi flies at each age point: *P < 0.05, **P < 0.01, **P < 0.001, and ****P < 0.0001. Error bars show standard error of the mean (SEM).

RNA was isolated from the thoraces of male *smooth* knockdown flies and their driver controls. Several genes of interest were evaluated using qPCR in knockdown flies from ages 6 days, 20 days, 30 days, and 50 days. We evaluated the following genes (the respective mammalian orthologs are in parentheses): *smooth* (*Hnrnpl*), *draper* (*Megf10*), *Serrate* (*Jag1/Jag2*), *rumi* (*Poglut1*), *Notch* (*Notch1‐4*), *misfire* (*Dysf*), and *Limpet* (*Fhl1/Fhl2*) normalized to housekeeping gene *Act5C* (*Actb*) levels. *Smooth* was knocked down at every time point. At Day 6, *Limpet* was upregulated, and no other genes had significant expression changes (Fig. 8B). At Day 20, all genes examined except *smooth* were significantly upregulated (Fig. 8C). At Day 30, *rumi* and *Notch* became downregulated (Fig. 8D). At Day 50, *misfire* became downregulated as well (Fig. 8E). PCR on cDNA was performed to look for potential splicing changes in *smooth* (exons 2–5), *draper* (exons 8–12), *Serrate* (exons 2–14), *Notch* (exons 6–8), and *rumi* (exons 1–2). PCRs confirmed *smooth* knockdown, but no splicing changes were observed between the evaluated exons (data not shown). Due to insufficient quantities of RNA obtained from the flies, nanopore long‐read transcriptome sequencing (RNAseq) could not be performed from these samples to evaluate other potential regulation or splicing changes.

## Discussion

HnRNP L is known to be important for muscle development [26, 27, 31], yet the range of its interactions with genes that underlie muscle function remains to be fully defined. Here, we demonstrate dysregulation of important signaling pathway genes and muscle function genes in the context of *Hnrnpl* knockdown, most notably at the myotube stage, as assessed using the Notch plate array. In addition, several muscle genes displayed altered splicing patterns at the myoblast stage.

*Hnrnpl* knockdown results in significant downregulation of *Mbnl2* and upregulation of *Mbnl1*, two splicing factors that are linked to myotonic dystrophy type 1 (DM1). MBNL1 and MBNL2 are both required for late myogenic maturation in human skeletal muscle cells, and knockout of either *Mbnl1* or *Mbnl2* in mice serves as a model for myotonic dystrophy [47, 48, 49]. In addition, hnRNP L and MBNL1 are reported to be binding partners [31]. Loss of *Mbnl2* was previously demonstrated to increase aberrant splicing [73]. Downregulation of *Mbnl2* likely exacerbates *Hnrnpl* knockdown‐mediated aberrant gene splicing and may thus contribute to the defects observed in *Hnrnpl* deficient C2C12 cells. *Mbnl2* compensates for *Mbnl1* deficiency, but it is unclear whether the converse is true [49, 74]. *Mbnl1* overexpression rescues the phenotype of human DM1 skeletal muscle stem cells [75]. Further studies could determine whether *Mbnl1* upregulation is a response to *Hnrnpl* deficiency or a compensatory mechanism to *Mbnl2* deficiency. Additionally, further studies could evaluate if other splicing changes found in the *Hnrnpl* knockdown condition are directly caused by the change in *Hnrnpl* expression levels or if the splicing changes are caused by other splicing factors affected by hnRNP L deficiency, such as Mbnl1 and Mbnl2.

The most downregulated gene in the *Hnnrpl* knockdown condition was *Stmn2*. *Stmn2* is an important regulator of motor and sensory neuron growth and repair. Deficiency of *Stmn2* in mice leads to motor deficits with denervation of the neuromuscular junctions [76, 77]. Previous work has found that overexpression of hnRNP L increases *Stmn2* expression levels [78]. Taken together, these findings suggest that *Hnrnp*l and *Stmn2* expression are correlated, and that knockdown of *Hnrnpl* affects *Stmn2‐*mediated function. Notably, the pathway enrichment results of the deseq2 analysis shows downregulation of genes involved in neuron projection development (*Ankrd1*, *Cfl1*, *Dpysl3*, *Fxn*, *H2‐K1*, *Hspb1*, *Map2k1*, *Marcks*, *Nefl*, *Obsl1*, *Rpl4*, *Serpinf1*, *Sgk1*, *Stmn2*, *Tlx2*, and *Tubb2b*) and regulation of dendrite morphogenesis (*Marcks*, *Obsl1*, *Sgk1*, and *Tlx2*). Additionally, individuals affected by DM1 often have central nervous system complications, and dendrite abnormalities have been noted in *Mbnl2* knockout mice [79]. Follow‐up studies *in vivo* may probe the potential effect of *Hnrnpl* deficiency on muscle innervation.

*Lamp2* was differentially spliced between *Hnrnpl* knockdown and scrambled control cells, with transcript variant 1 being downregulated and transcript variant 2 being upregulated. The protein product of *LAMP2A* (the human ortholog of mouse *Lamp2* transcript variant 1) serves as a receptor and a protein channel on lysosomes for chaperone‐mediated autophagy (CMA), a highly selective form of autophagy that imports cytosolic proteins bearing a KFERQ‐like motif to lysosomes for degradation. Blockage of LAMP2A inhibits CMA. CMA dysfunction has been linked to cardiovascular diseases, aging, neurodegenerative diseases, cancer, and metabolic syndrome. LAMP2A is expressed in multiple tissues and organs, including at low levels in skeletal muscle; LAMP2B is highly expressed in skeletal muscle, cardiac muscle, and brain [80]. The protein product of *LAMP2B* (the human ortholog of mouse *Lamp2* transcript variant 2) promotes autophagic fusion, which targets cargo for degradation in the endosome or lysosome. This is important for the removal of unhealthy mitochondria [81]. Thus, increased *Lamp2* variant 2 levels may reflect an accumulation of defective mitochondria.

The enriched pathways of differentially expressed genes indicate that *Hnrnpl* knockdown C2C12 cells experience increased apoptosis. This agrees with previous work indicating that *HNRNPL* knockdown in human primary myotubes resulted in increased caspase‐3 induced apoptosis, approximately 165‐fold more so than the control myotubes [31]. Knockdown of *HNRNPL* in human prostate cancer cell lines also increased apoptosis [82]. While we did not see reduced numbers of *Hnrnpl* knockdown cells in our proliferation assay, we hypothesize that apoptotic signals will be more consequential during differentiation. Notably, we previously found no change in myoblast proliferation and increased myotube apoptosis when *HNRNPL* is knocked down [31].

The enriched pathways also indicated that *Hnrnpl* knockdown cells were experiencing oxidative stress. The most common cause of oxidative stress in cells is aberrant mitochondrial respiration/ mitochondrial damage [83]. This, along with the increase in *Lamp2* transcript variant 2, suggests that *Hnrnpl* deficiency may be linked to mitochondrial dysfunction; however, caspase‐3 is activated by both intrinsic and extrinsic death pathways in apoptosis [84], so we cannot conclude with certainty that mitochondrial dysfunction is the cause of the oxidative stress and increased apoptosis in the *Hnrnpl* knockdown condition without further validation. Although oxidative stress is known to increase CMA through transcriptional upregulation of *LAMP2A* [85], we observed diminished expression of *Lamp2* transcript variant 1. *Lamp2* splicing patterns may be downstream of *Hnrnpl* expression rather than oxidative stress, and *Hnrnpl* and *Lamp2* transcript variant 1 expression levels could potentially be directly correlated. The downregulation of *Lamp2* transcript variant 1 would likely lead to a reduction in CMA. Reduced CMA, which is linked to neurodegeneration [86], might also support the hypothesis that muscle denervation may be correlated with *Hnrnpl* deficiency. The increased expression of *Lamp2* transcript variant 2 may induce excess autophagic fusion and removal of mitochondria, leading in turn to cellular distress. Future studies may further probe this possibility.

*Fhl1* is a known muscular dystrophy gene which was downregulated on deseq2 analysis in the setting of *Hnrnpl* knockdown. KyoT1, also known as FHL1A in humans, is the predominant isoform of FHL1, localizing to the I‐band of mature skeletal muscle and regulating sarcomere assembly [87]. Numerous pathogenic variants in *FHL1* specifically affect the FHL1A isoform [88]. KyoT2 and KyoT3 both localize within the nucleus and contain an RBP‐J‐binding motif. These two isoforms bind to RBP‐J and are potent inhibitors of Notch signaling; it is postulated that KyoT2, and potentially KyoT3, promote muscle differentiation by blocking the inhibitory effect that Notch has on myogenesis [62, 89, 90]. Downregulation of these isoforms could contribute to decreased myogenesis through lack of Notch signaling inhibition. KyoT3 binds to and inhibits pro‐apoptotic proteins in muscle cells, protecting them from apoptosis [91]. The decreased abundance of KyoT3 could have contributed to increased apoptotic signaling in our *Hnrnpl* knockdown cells.

*Dtna* underwent differential splicing in the setting of *Hnrnpl* knockdown, with transcript variant 3/X35 being downregulated and transcript variant X39 being upregulated. The human gene *DTNA* has 21 coding exons that encode three major isoforms: α‐DB1, α‐DB2, and α‐DB3, ending at exon 21, exon 17B, and exon 11B respectively. Three variable regions are subject to alternative splicing within the major isoforms. Alternative splicing of *DTNA* variable region 2 is dysregulated in the muscles of individuals with myotonic dystrophy [92, 93]. In mouse, transcript variant 3 and predicted transcript variant X35 both encode α‐DB1. Predicted transcript variant X39 encodes α‐DB3.

Our data show a downregulation of transcripts encoding *Dtna* isoform α‐DB1 and an upregulation of transcripts encoding *Dtna* isoform α‐DB3. α‐DB1 and α‐DB3 are both expressed early in the development of C2C12 cells. α‐DB1 is expressed predominantly in skeletal muscle and the heart at the neuromuscular junction, and binds utrophin, dystrophin, and syntrophin. α‐DB1 is a key regulator of synaptic maturation. α‐DB3 is predominantly expressed in skeletal muscle, but this shorter isoform lacks a dystrophin binding region. Although it lacks this binding region, α‐DB3 partially rescues *Dtna* knockout and becomes integrated into the dystrophin‐glycoprotein complex [94, 95, 96, 97]. Downregulation of α‐DB1 may also contribute to the denervation of the muscle, similar to downregulation of *Lamp2* transcript variant 1 and *Stmn2*. The upregulation of transcripts encoding α‐DB3 may partially compensate for loss of α‐DB1; additional studies would be needed to confirm this.

The *Drosophila* data confirmed that many genes within the same pathways are dysregulated in the setting of *smooth* and *Hnrnpl* knockdown. However, some genes display divergent dysregulation. For example, *Limpet* was consistently upregulated in *smooth* knockdown flies, while the ortholog *Fhl1/KyoT* was downregulated in *Hnrnpl* knockdown C2C12 myoblasts. Limpet is the closest fly ortholog to KyoT2 as they share a similar LIM domain; however, in contrast to KyoT2 in mammalian cells, Limpet does not appear to act as a Notch repressor in invertebrates. Mechanisms of Notch repression are present in both vertebrates and higher arthropods; however, they may recruit different partners [98]. Knockout of *Limpet* leads to a cardiac phenotype, and this factor is a repressor of Wnt signaling in muscle rather than Notch signaling [99, 100], although cross‐talk between these pathways is known [101, 102]. Wnt signaling is essential for myogenesis [103, 104], so increased expression of *Limpet* may affect muscle development. *Serrate* was also consistently upregulated in the flies, while its homologs *Jag1* and *Jag2* were both downregulated in the C2C12 cells. *Serrate* is a Notch ligand that either activates Notch (via a trans effect from adjacent cells) or inhibits Notch (via a cell‐autonomous/cis effect) [105, 106]. Further studies could investigate whether *Serrate* is upregulated on cells expressing Notch or upregulated on cells lacking Notch.

Our findings suggest that hnRNP L contributes to the regulation of Notch signaling and myoblast differentiation, with the latter corresponding to results from prior studies [26, 27]. Of note, hnRNP I, which shares significant homology with hnRNP L, is a known Notch inhibitor [107] and can partner with hnRNP L [108]. Notch signaling diminishes as myoblasts differentiate, but excessive augmentation or suppression of Notch signaling disrupts the complex processes involved [109]. Our findings of impaired mouse myoblast differentiation in the setting of *Hnrnpl* knockdown and impaired late *Drosophila* myogenesis in the setting of *smooth* inhibition align with our prior work in *HNRNPL* knockdown human myoblasts [31]. We found that *Hnrnpl* knockdown is associated with downregulation of the Notch co‐repressor KyoT2, yet we also observed that several Notch genes were downregulated, including transcriptional repressor *Hey1*, which inhibits myogenesis. Despite this, downregulation of *Hey1* in isolation does not stimulate myogenesis, as other Notch signaling components inhibit this process [110]. Binding sites for hnRNP L have been noted on the mRNA of *JAG2* [2], which encodes a canonical Notch ligand and muscular dystrophy gene. The selective downregulation of *Jag2* in the myoblast stage but not in the myotube stage in our studies of *Hnrnpl* knockdown is an indication of the delicate temporal regulation of Notch signaling during myogenesis.

**Fig. 9:**
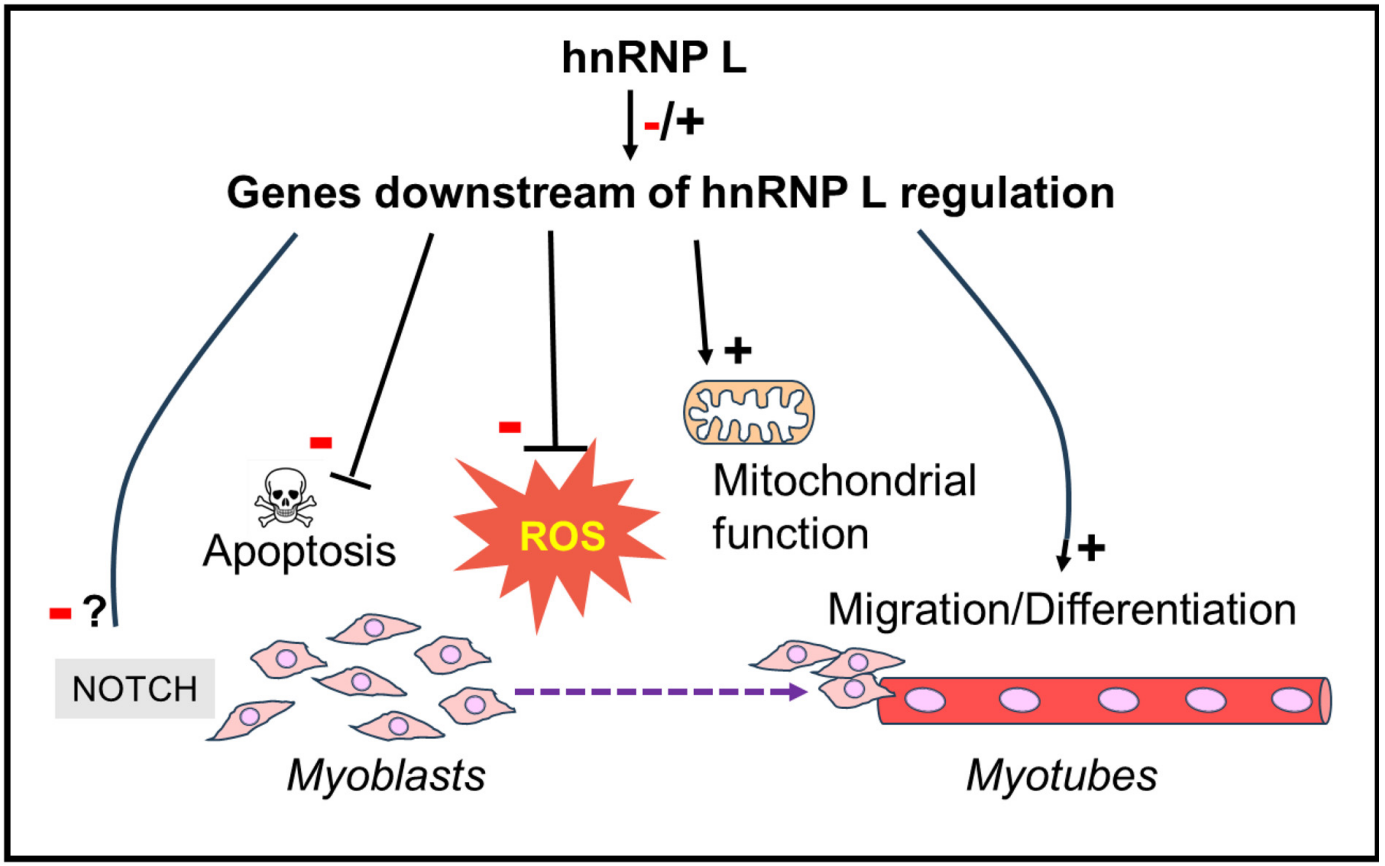
Diagram illustrating the regulatory contributions of hnRNP L to myogenesis.

In B cells, hnRNP L contributes to the transition from a resting to activated state. Downregulation of *Hnrnpl* in B cells leads to mitochondrial dysfunction, accumulation of reactive oxygen species (ROS), and increased apoptosis [111]. We found similar consequences of *Hnrnpl* downregulation in myoblasts (Fig. 9).

Our comparison of the nanopore RNAseq data with PCR and qPCR analyses provides a window into the capabilities of nanopore RNAseq to detect changes in splicing patterns. Our nanopore data analyses correlated well with PCR and/or qPCR data for *Lamp2*, *Tpm1*, and *Fhl1*, while our nanopore data analyses did not detect the splice differences for *Dtna*. The read depth of the nanopore RNAseq was notably lower for *Dtna* than for the other genes that we examined in depth, which provides an opportunity to take a preliminary look at what read depth might be needed to detect transcript variants of particular frequencies. The comparison of splicing changes identified by bioinformatic analyses versus visual inspection suggests that a minimum nanopore read depth of 10 is necessary to routinely identify differential splicing patterns that consistently vary by more than 10% to 20% across replicates. Future studies could include comparisons of nanopore reads with validation by PCR and/or qPCR for additional genes for a more comprehensive determination of the read depths needed to detect specific isoform frequencies.

A technical limitation of our study is the tendency of nanopore RNAseq to favor the 3′ end of the gene, rather than the whole transcript, reducing our ability to detect splicing changes that occur toward the 5′ end of the gene and reducing the proportion of reads that could be assigned to specific transcript variants. This issue has been reported previously [112]. Our choice of direct RNA sequencing, a PCR‐free method, is beneficial because no PCR bias is introduced, but it limited our ability to detect transcripts with low expression levels such as many of the Notch genes. A new nanopore RNA‐004 kit promises to improve transcriptome‐wide coverage and transcript variant identification, but it was not available when we conducted the sequencing experiments for the current study.

We were only able to generate two replicates of nanopore RNAseq for each group, but we countered this limitation by using the deseq2, isoformswitchanalyzer, and dexseq analytic tools, which yielded statistically significant calculations and by analyzing at least three independent replicates for our PCR and qPCR studies. Comparisons of fold changes of the Notch plate array genes between deseq2 and qPCR showed a moderate positive correlation (Fig. S2); even though the majority of the changes in gene expression were not significant in deseq2, the fold changes were still similar to what was seen in qPCR. While deseq2 predictions can be inaccurate when the adjusted *P*‐value is above the 0.05 cut off (Table 1), deseq2 estimated most fold changes with reasonable accuracy even with low replicates and low coverage (Fig. S2).

Due to insufficient quantities of escaper flies, we were unable to perform nanopore long read whole transcriptome sequencing or assess potential splicing changes in this model. Defining the downstream mechanistic effects of individual transcriptome changes on developing muscle cells and flies would be of great interest, but those experiments are beyond the scope of the current study.

We conclude that the splicing factor hnRNP L is critical for muscle development, potentially due to downstream alterations in expression levels and splicing patterns of the genes studied here. Our work indicates that hnRNP L may play a role in the mechanisms of several inherited muscle diseases. Future studies could examine the precise splice alterations leading to nonsense mediated decay, as well as downstream effects on protein isoforms and cellular functions. In addition, it might be of interest to investigate whether enhancing hnRNP L expression may provide a potential therapeutic strategy for muscle disease.

## Conflict of interest

The authors declare no conflict of interest.

## Author contributions

HRL, MG, and PBK designed the experiments, analyzed the data, and wrote the manuscript with input and editing from ALD, CAP, CCB, and ID. C2C12 knockdown cells were generated by MG with the help of NMW, where MG performed experiments and analyzed the data. JT assisted MG with PCR analysis of C2C12 RNA. HRL performed RNAseq and subsequent computational and molecular analysis on the C2C12 RNA. ID developed the knockdown flies and tested varying drivers, and ALD performed *Drosophila* gene expression analysis. All authors reviewed a draft of the manuscript and provided input.

## Supporting information


**Fig. S1.** Cell counts from the proliferation assay on Hnrnpl knockdown cells.
**Fig. S2.** Linear regression of fold changes found from the Notch plate array genes when comparing the qPCR fold changes found in myoblasts on the *X* axis and the fold changes found by deseq2 from the nanopore RNA sequencing of myoblasts on the *Y* axis.

**Table S1.** qPCR probes used for relative gene expression assays.
**Table S2.** PCR primers used on C2C12 cDNA to confirm splicing changes.
**Table S3.** Full Notch plate array qPCR results, with a comparison to the deseq2 results of the same genes using the Wald test and local fit.
**Table S4.** Results of Nanopore RNA sequencing.
**Table S5.** Number of significant genes found by deseq2 using different analysis parameters.
**Table S6.** Significant deseq2 results of Wald test, parametric fit.
**Table S7.** Significant deseq2 results of Wald test, local fit.
**Table S8.** Significant deseq2 results of LRT, parametric fit.
**Table S9.** Significant deseq2 results of LRT, local fit.
**Table S10.** Comparison of significant deseq2 results across analytical methods.
**Table S11.** deseq2 results of all the hnRNP genes using the Wald test and local fit.
**Table S12.** Significant IsoformSwitchAnalyzeR results.
**Table S13.** Significant DEXseq results.
**Table S14.** Comparison of significant IsoformSwitchAnalyzeR results and significant DEXseq results.
**Table S15.** Nanopore coverage of genes with splicing changes found.
**Table S16.** DEXseq results of *Fhl1*.
**Table S17.** IsoformSwitchAnalyzeR and DEXseq results of *Dtna*.

**Data S1.** R scripts used for nanopore RNAseq analysis (PDF).

## Data Availability

The RNAseq data that support the findings of this study are available in NCBI Gene Expression Omnibus (GEO) at https://www.ncbi.nlm.nih.gov/geo/query/acc.cgi?acc=GSE302000, reference number GSE302000. See the Supporting Information section regarding Supplementary Materials.
