# Supplementary material for: The impact of Hnrnpl deficiency on transcriptional patterns of developing muscle cells
Source: FEBS Open Bio. 2025 Sep 13;16(1):178–98. doi: 10.1002/2211-5463.70117 (PMC12767766; doi:10.1002/2211-5463.70117)
Supplement: Supplementary file 1 — Fig. S1. Cell counts from the proliferation assay on Hnrnpl knockdown cells. Fig. S2. Linear regression of fold changes found from the Notch plate array genes when comparing the qPCR fold changes found in myoblasts on the X axis and the fold changes found by deseq2 from the nanopore RNA sequencing of myoblasts on the Y axis. [file FEB4-16-178-s002.docx]

**Figure S1**. Cell counts from the proliferation assay on Hnrnpl knockdown cells. 5000 C2C12 myoblasts were seeded on day 0. The cells were stained with trypan blue using a 1:1 dilution on days 1, 2, and 3, and then counted using a hemocytometer. Results are from N=2 replicates. Unpaired t-tests were used for significance, but no significance was found. Error bars show standard error of the mean (SEM).

**Figure S2**. Linear regression of fold changes found from the Notch plate array genes when comparing the qPCR fold changes found in myoblasts on the X axis and the fold changes found by DESeq2 from the nanopore RNA sequencing of myoblasts on the Y axis. Each dot represents a gene from the Notch plate array, found in Table S3. 69 total genes are plotted. The following genes were removed due to having either undetermined qPCR fold changes, and/or a baseMean of 0 in DESeq2: *Dll4*, *Dtx1*, *Cdkn2a*, *Il1b*, *Il6*, *Gbp2*, *Gli1*, *Hoxb4*, *Tnfa* (*Tnf*), *Ifng*, *Il17b*, *Il2ra*, *Mfng*, *Mmp7*, *Notch1*, *Notch4*, *Pax5*, *Ptcra*, *Shh*, *Myog*, *Myh2*, *Stat6*, *Dll3*, *Pax3*, *Pax7*, and *Myod1*. The gene *Fosl1* was also removed for being an outlier in its qPCR data. The Pearson r= 0.5215, with a 95% confidence interval from 0.3249 to 0.6749. The two tailed p-value was <0.0001.
