# Supplementary material for: The impact of Hnrnpl deficiency on transcriptional patterns of developing muscle cells
Source: FEBS Open Bio. 2025 Sep 13;16(1):178–98. doi: 10.1002/2211-5463.70117 (PMC12767766; doi:10.1002/2211-5463.70117)
Supplement: Supplementary file 3 — Data S1. R scripts used for nanopore RNAseq analysis (PDF). [file FEB4-16-178-s003.pdf]

```
#DESeq2 script ########################################################
#set working directory to where the htseq count files are; htseq performed on .bam file
mapped to mm39 using splice-aware minimap2
setwd("/home/pkang/shared/nanoporeData/Deseq2-opt/Hnrnpl/")

#BiocManager::install("DESeq2") #only install the first time
library(DESeq2)
library(ggplot2)
Sample_names <- data.frame("Scrambled control 1", "Scrambled control 2", "Hnrnpl
knockdown 1", "Hnrnpl knockdown 2")
Count_files <- c("HNRNPL_scrambled_RNA_20220822.ens_count", "HNRNPL-scr-
RNA2_20221005.ens_count","HNRNPL_knockdown_RNA_20220822.ens_count","HNRNPL-kd-
RNA2_20221005.ens_count")
Condition <- c("Scrambled control", "Scrambled control", "Hnrnpl knockdown", "Hnrnpl
knockdown")

deseqcols <- rbind(Sample_names, Count_files, Condition)
sampleTable <- as.data.frame(t(deseqcols))
colnames(sampleTable) <- c("Sample name", "Count file name", "Condition")

#put into table for DESeq2 to use, can input using the count file names if you're in the correct
directory
Deseq2datatable <- DESeqDataSetFromHTSeqCount(sampleTable, directory = ".", design = ~
Condition, ignoreRank = FALSE)

#####################
#trying different statistical tests and fit types; start with default (wald test, parametric fit)
dds_wp <- DESeq(Deseq2datatable) #default design, test = Wald, fitType = parametric
res_wp <- results(dds_wp, contrast = c("Condition", "Hnrnpl knockdown", "Scrambled control"),
alpha = 0.05)
write.csv(res_wp, file = "deseq2_results_all-genes-Hnrnpl-Wald-parametric-alpha0.05-
final.csv")
sigs_wp <- na.omit(res_wp)
sigs_wp <- sigs_wp[sigs_wp$padj < 0.05,] #filters out the genes with a p-value less than 0.05
df_wp <- as.data.frame(sigs_wp) #119 genes
write.csv(sigs_wp, file = "deseq2_results_sig-genes-Hnrnpl_Wald-parametric-alpha0.05.csv")
df_wp.top <- df_wp[ (df_wp$baseMean > 10) & (abs(df_wp$log2FoldChange) > 0.5),] #115
genes
df_wp.top <- df_wp.top[order(df_wp.top$log2FoldChange, decreasing = TRUE),]
write.csv(df_wp.top, file = "deseq2_results_Hnrnpl-wald-parametric-p0.05-filtered.csv")

#Wald test local fit
dds_wl <- DESeq(Deseq2datatable, fitType = c("local")) #must specify local, default is
parametric, local gets more genes
```

```
res_wl <- results(dds_wl, contrast = c("Condition", "Hnrnpl knockdown", "Scrambled control"),
alpha = 0.05)
write.csv(res_wl, file = "deseq2_results_all-genes-Hnrnpl-Wald-local-alpha0.05-final.csv")
sigs_wl <- na.omit(res_wl)
sigs_wl <- sigs_wl[sigs_wl$padj < 0.05,] #filters out the genes with a p-value less than 0.05
df_wl <- as.data.frame(sigs_wl) #237 genes
write.csv(sigs_wl, file = "deseq2_results_sig-genes-Hnrnpl_Wald-local-alpha0.05.csv")
df_wl.top <- df_wl[ (df_wl$baseMean > 10) & (abs(df_wl$log2FoldChange) > 0.5),] #228 genes
df_wl.top <- df_wl.top[order(df_wl.top$log2FoldChange, decreasing = TRUE),]
write.csv(df_wp.top, file = "deseq2_results_Hnrnpl-wald-parametric-p0.05-filtered.csv")

#LRT, parametric fit
dds_lp <- DESeq(Deseq2datatable, test = c("LRT"), fitType = c("parametric"), full =
design(Deseq2datatable), reduced = ~1)
res_lp <- results(dds_lp, contrast = c("Condition", "Hnrnpl knockdown", "Scrambled control"),
alpha = 0.05, test = c("LRT"))
write.csv(res_lp, file = "deseq2_results_all-genes-Hnrnpl-LRT-parametric-alpha0.05-final.csv")
sigs_lp <- na.omit(res_lp)
sigs_lp <- sigs_lp[sigs_lp$padj < 0.05,] #filters out the genes with a p-value less than 0.05
df_lp <- as.data.frame(sigs_lp) #117 genes
write.csv(sigs_lp, file = "deseq2_results_sig-genes-Hnrnpl_LRT-parametric-alpha0.05.csv")
df.top_lp <- df_lp[ (df_lp$baseMean > 10) & (abs(df_lp$log2FoldChange) > 0.5),] #112 genes
df.top_lp <- df.top_lp[order(df.top_lp$log2FoldChange, decreasing = TRUE),]
write.csv(df_wp.top, file = "deseq2_results_Hnrnpl-wald-parametric-p0.05-filtered.csv")

#LRT, local fit
dds_ll <- DESeq(Deseq2datatable, test = c("LRT"), fitType = c("local"), full =
design(Deseq2datatable), reduced = ~1)
res_ll <- results(dds_ll, contrast = c("Condition", "Hnrnpl knockdown", "Scrambled control"),
alpha = 0.05, test = c("LRT"))
write.csv(res_lp, file = "deseq2_results_all-genes-Hnrnpl-LRT-parametric-alpha0.05-final.csv")
sigs_ll <- na.omit(res_ll)
sigs_ll <- sigs_ll[sigs_ll$padj < 0.05,] #filters out the genes with a p-value less than 0.05
df_ll <- as.data.frame(sigs_ll) #237 genes
write.csv(sigs_lp, file = "deseq2_results_sig-genes-Hnrnpl_LRT-parametric-alpha0.05.csv")
df.top_ll <- df_ll[ (df_ll$baseMean > 10) & (abs(df_ll$log2FoldChange) > 0.5),] #222 genes
df.top_ll <- df.top_ll[order(df.top_ll$log2FoldChange, decreasing = TRUE),]
write.csv(df_wp.top, file = "deseq2_results_Hnrnpl-wald-parametric-p0.05-filtered.csv")

#continuing with Wald test, local fit
#graphs
r_normalized_data <- rlog(Deseq2datatable, blind = TRUE, fitType = "local") #specify local
instead of parametric, better fit, more similar within groups
plotPCA(r_normalized_data, intgroup = "Condition", ntop = 500, returnData = FALSE)
```

```
#volcano plot
# https://biostatsquid.com/volcano-plots-r-tutorial/
library(tidyverse)
library(RColorBrewer)
library(ggrepel)

fulldata <- na.omit(res_wl) #remove all genes without p-adjusted values
fulldata <- as.data.frame(fulldata) #8507 detected genes

# Add a column to the data frame to specify if they are UP- or DOWN- regulated
fulldata$diffexpressed <- "NO"
fulldata$diffexpressed[fulldata$log2FoldChange > 0.5 & fulldata$padj < 0.05] <- "UP"
fulldata$diffexpressed[fulldata$log2FoldChange < -0.5 & fulldata$padj < 0.05] <- "DOWN"
mytitle <- expression(paste("Differentially expressed genes in ", italic("Hnrnpl"), " knockdown
cells"))

ggplot(data = fulldata, aes(x = log2FoldChange, y = -log10(padj), col = diffexpressed)) +
  geom_vline(xintercept = c(-0.5, 0.5), col = "gray", linetype = 'dashed') +
  geom_hline(yintercept = -log10(0.05), col = "gray", linetype = 'dashed') + #should be 0.055
instead, if that's the p-value cut off I use
  geom_point(size = 2) +
  scale_color_manual(values = c("#bb0c00", "grey","#00AFBB"), # to set the colours of our
variable
                     labels = c("Downregulated", "Not significant", "Upregulated")) + # to set the labels
in case we want to overwrite the categories from the dataframe (UP, DOWN, NO)
  labs(color = 'Expression', #legend_title,
       x = expression("log"[2]*"FC"), y = expression("-log"[10]*"adjusted p-value")) +
  ggtitle(mytitle) # Plot title

#heat map
#tutorial here:
https://github.com/mousepixels/sanbomics_scripts/blob/main/tutorial_complex_Heatmap.Rm
d
#youtube: https://www.youtube.com/watch?v=ht1r34-ifVI
rlog_out <- rlog(dds_wl, blind=FALSE) #get normalized count data from dds object
mat<-assay(rlog_out)[rownames(df_wl.top), sampleTable$`Sample name`] #sig genes x
samples; #this has the rlog value of the gene expression for the individual samples
base_mean <- rowMeans(mat)
mat.scaled <- t(apply(mat, 1, scale)) #center and scale each column (Z-score) then transpose
colnames(mat.scaled)<-colnames(mat)

#next commands keep top and bottom 15 genes
num_keep <- 15
```

```
rows_keep <- c(seq(1:num_keep), seq((nrow(mat.scaled)-num_keep+1), nrow(mat.scaled)) )
l2_val <- as.matrix(df_wl.top[rows_keep,]$log2FoldChange) #getting log2 value for each gene
we are keeping
mean <- as.matrix(df_wl.top[rows_keep,]$baseMean) #getting mean value for each gene we
are keeping

colnames(l2_val)<-"log2FC"
colnames(mean)<-"AveExpr"

#name for rows; need to manually input these
top15 <- c("Mgl2", "Platr3", "Ptn", "Fgf7", "Sh2d1b1", "Cavin2", "Igsf11", "Crybg1", "Lgr6",
"Podnl1", "Sema3a", "Ephx1", "Pparg", "Rin2", "Id2",
        "Cdh15", "Gpx3", "Mcam", "Tubb3", "Fhl1", "Sox11", "Wnt7b", "Sulf2", "Socs2", "Ildr2",
"Pknox2", "Ptgis", "Mmp15", "Il11", "Stmn2")

#BiocManager::install("ComplexHeatmap") #only install this once
library(ComplexHeatmap)
library(RColorBrewer)
library(circlize)
#maps values between b/w/r for min and max l2 values
col_log2FC <- colorRamp2(c(min(l2_val),0, max(l2_val)), c("blue", "white", "red"))
#maps between 0% quantile, and 75% quantile of mean values --- 0, 25, 50, 75, 100
col_AveExpr <- colorRamp2(c(quantile(mean)[1], quantile(mean)[4]), c("white", "red"))
ha <- HeatmapAnnotation(summary = anno_summary(gp = gpar(fill = 2),
                                height = unit(2, "cm")))
h1 <- Heatmap(mat.scaled[rows_keep,], cluster_rows = F,
          column_labels = colnames(mat.scaled), name="Z-score",
          cluster_columns = T)

h2 <- Heatmap(l2_val, row_labels = top15,
          cluster_rows = F, name="log2FC", top_annotation = ha, col = col_log2FC,
          cell_fun = function(j, i, x, y, w, h, col) { # add text to each grid
            grid.text(round(l2_val[i, j],2), x, y)
          })

h3 <- Heatmap(mean, row_labels = top15,
          cluster_rows = F, name = "AveExpr", col=col_AveExpr,
          cell_fun = function(j, i, x, y, w, h, col) { # add text to each grid
            grid.text(round(mean[i, j],2), x, y)
          })

h<-h1+h2+h3
h
```

```
#IsoformSwitchAnalyzeR script ##################################################
#before starting R script:

#before doing minimap and nanocount, you need make a fasta reference file with only the gencode transcript_ids
#first unzip the gencode fasta file (downloaded from https://www.gencodegenes.org/mouse/) using 'gunzip gencode.vM33.transcripts.fa.gz'
#then perform the following command:
#awk 'BEGIN {FS =OFS="|" } /^>/ {print $1 ; next }1' gencode.vM33.transcripts.fa > gencode_transcript_id.fa
#this kept only the first field, which was the gencode transcript ID, and kept the sequence
#then do minimap using this as the reference (minimap to transcriptome, don't do splice aware to genome), and do nanocount on the resulting .bam file
#minimap2 -t 4 -ax map-ont -N 10 gencode_transcript_id.fa reads.fastq | samtools view -bh > aligned_reads.bam
#NanoCount -i aligned_reads_sorted.bam -b output/aligned_reads_selected.bam --extra_tx_info -o output/tx_counts.tsv
#then read in the .tsv files from nanocount into R and use following script

#set working directory to where files are
setwd("/home/pkang/shared/nanoporeData/Deseq2-opt/IsoformSwitchAnalyzeR_Hnrnpl")

counts_scr1 <- read.table(file = 'scrambled-RNA1-gencode.tsv', sep = '\t', header = TRUE)
counts_scr2 <- read.table(file = 'scrambled-RNA2-gencode.tsv', sep = '\t', header = TRUE)
counts_kd1 <- read.table(file = 'Hnrnpl-kd-RNA1-gencode.tsv', sep = '\t', header = TRUE)
counts_kd2 <- read.table(file = 'Hnrnpl-kd-RNA2-gencode.tsv', sep = '\t', header = TRUE)

rownames(counts_scr1) <- counts_scr1[,1]
rownames(counts_scr2) <- counts_scr2[,1]
rownames(counts_kd1) <- counts_kd1[,1]
rownames(counts_kd2) <- counts_kd2[,1]

#nanocount .tsv files are in order of estimated counts, not gene names
#need to put the tables in the same order so they can be combined

transcript_order <- c(counts_scr1$transcript_name)
ordered_scr2 <- counts_scr2[transcript_order , ]
ordered_kd1 <- counts_kd1[transcript_order , ]
ordered_kd2 <- counts_kd2[transcript_order , ]
#all tables are in the row order of scrambled 1 now

est_counts <- cbind(counts_scr1$transcript_name, counts_scr1$est_count,
ordered_scr2$est_count, ordered_kd1$est_count, ordered_kd2$est_count)
```

```
colnames(est_counts) <- c("isoform_id", "scr1", "scr2", "kd1", "kd2") #column must be named
isoform_id for it import data properly
write.csv(est_counts, file = "nanocount-estimated-counts-transcript-id.csv")

count_matrix <- read.csv("nanocount-estimated-counts-transcript-id.csv", header=T)
count_matrix=count_matrix[ ,-1] #gets rid of the extra column

myDesign <- data.frame(sampleID = c("scr1", "scr2", "kd1", "kd2"),condition =
c("scrambled_control", "scrambled_control", "Hnrnpl_knockdown", "Hnrnpl_knockdown"))
#sample ID must match the column names in the count matrix

comparisons <- data.frame(condition_1 = c("scrambled_control"), condition_2 =
c("Hnrnpl_knockdown")) #specify condition 1 as the baseline control

#if (!require("BiocManager", quietly = TRUE))
# install.packages("BiocManager")
#BiocManager::install("IsoformSwitchAnalyzeR")

library(IsoformSwitchAnalyzeR)

aSwitchList <- importRdata(
  isoformCountMatrix   = count_matrix,
  designMatrix         = myDesign,
  isoformExonAnnoation = "gencode.vM33.chr_patch_hapl_scaff.annotation.gtf", #download
and unzip from gencode
  isoformNtFasta       = "gencode_transcript_id.fa",
  showProgress = FALSE,
  comparisonsToMake = comparisons
)

write.csv(aSwitchList$isoformFeatures, "ISA-all-results-final.csv")

SwitchListFiltered <- preFilter(
  switchAnalyzeRlist = aSwitchList,
  geneExpressionCutoff = 10, #10 to be consistent with DESeq2 and DEXseq
  isoformExpressionCutoff = 5, #will keep at 5 like Gleeson et al.
  removeSingleIsoformGenes = TRUE)


# tests for differential isoform usage
SwitchListAnalyzed <- isoformSwitchTestDEXSeq(
  switchAnalyzeRlist = SwitchListFiltered,
  reduceToSwitchingGenes=TRUE,
  reduceFurtherToGenesWithConsequencePotential = FALSE,
```

```
  alpha = 0.05,
  dIFcutoff = 0.1,
  onlySigIsoforms = FALSE
)


extractSwitchSummary(SwitchListAnalyzed)
write.csv(SwitchListAnalyzed$isoformFeatures, "differential_isoform_usage-final.csv")

#DEXSeq script ####################################################################
#set working directory to where the files are; uses the .bam files from minimap2, using splice-
aware mapping to mm39
setwd("/home/pkang/shared/nanoporeData/Deseq2-opt/DEXseq_Hnrnpl")

#BiocManager::install(c("DEXSeq"))
#BiocManager::install(c("GenomicFeatures"))

#help links:
#https://bioconductor.org/packages/release/bioc/vignettes/DEXSeq/inst/doc/DEXSeq.html#1_
Overview
#DEXSeq manual:
#https://bioconductor.org/packages/release/bioc/manuals/DEXSeq/man/DEXSeq.pdf

library(DEXSeq)
library(GenomicFeatures)

txdb <- makeTxDbFromGFF("gencode.vM30.primary_assembly.annotation.gtf.gz",
format="gtf")

flattenedAnnotation = exonicParts(txdb, linked.to.single.gene.only=TRUE)
names(flattenedAnnotation) = sprintf("%s:E%0.3d", flattenedAnnotation$gene_id,
flattenedAnnotation$exonic_part)

library(GenomicAlignments) #also loads Rsamtools with it

Scr1 <- BamFile("scrambled_RNA1.sorted.bam", index="scrambled_RNA1.sorted.bam.bai")
Scr2 <- BamFile("scrambled_RNA2.sorted.bam", index="scrambled_RNA2.sorted.bam.bai")
KD1 <- BamFile("Hnrnpl_kd_RNA1.sorted.bam", index="Hnrnpl_kd_RNA1.sorted.bam.bai")
KD2 <- BamFile("Hnrnpl_kd_RNA2.sorted.bam", index="Hnrnpl_kd_RNA2.sorted.bam.bai")
bamFiles <- c(Scr1, Scr2, KD1, KD2)
bamFiles = BamFileList(bamFiles) #need to convert to the right form

#Must use Big Mem resources (32 cores - 500Gb), or this command will fail; takes a few
minutes to complete
```

```
se <- summarizeOverlaps(flattenedAnnotation, bamFiles, singleEnd=TRUE, ignore.strand=TRUE,
inter.feature=FALSE)
colData(se)$sampleName = factor(c("Scrambled_1", "Scrambled_2", "Hnrnpl_knockdown_1",
"Hnrnpl_knockdown_2"))
colData(se)$condition = factor(c("scrambled_control", "scrambled_control",
"Hnrnpl_knockdown", "Hnrnpl_knockdown"))

dxd <- DEXSeqDataSetFromSE(se, design= ~ sample + exon + condition:exon) #default design

dxd = estimateSizeFactors(dxd)

dxd_loc = estimateDispersions(dxd, fitType = c("local")) #local was best fit for default design
plotDispEsts(dxd_loc)

dxd_loc = testForDEU(dxd_loc, fullModel = ~sample + exon + condition:exon, reducedModel =
~sample + exon, fitType = c("DESeq2"))
#default design, testing for differential exon usage

dxd_loc = estimateExonFoldChanges(dxd_loc, fitExpToVar="condition", denominator =
"scrambled_control")
dxr1 = DEXSeqResults(dxd_loc)

sigs <- na.omit(dxr1)
sigs #94975 rows and 13 columns
sigs_sav = as.data.frame(sigs)
sigs_sav <- apply(sigs_sav,2,as.character)
write.csv(sigs_sav, file = "DEXSeq-results-all-detected-exons.csv")

dxr1_0.05 <- sigs[sigs$padj < 0.05,]
dxr1_0.05 #275 rows and 13 columns
dxr1_0.05 = as.data.frame(dxr1_0.05)
dxr1_0.05 <- apply(dxr1_0.05,2,as.character)
write.csv(dxr1_0.05, file = "DEXSeq-results-padj0.05.csv")

#filtering
dxr_fil <- dxr1_0.05[ (dxr1_0.05$exonBaseMean > 10) &
(abs(dxr1_0.05$log2fold_Hnrnpl_knockdown_scrambled_control) > 0.5),] #132 exons
dxr_fil <- dxr_fil[order(dxr_fil$log2fold_Hnrnpl_knockdown_scrambled_control, decreasing =
TRUE),]
dxr_fil = as.data.frame(dxr_fil)
dxr_fil <- apply(dxr_fil,2,as.character)
write.csv(dxr_fil, file = "DEXseq-results-filtered.csv")
```
